# Gut microbiota remodeling exacerbates neuroinflammation and cognitive dysfunction via the microbiota-gut-brain axis in prenatal VPA-exposed C57BL/6 mice offspring

**DOI:** 10.3389/fimmu.2025.1633680

**Published:** 2025-08-13

**Authors:** Zhaoming Liu, Caixia Wu, Zhaojian Sun, Zuoxian Lin, Yirong Sun, Nouman Amjad, Muhammad Majid, Rajesh Basnet, Zhiyuan Li

**Affiliations:** ^1^ Guangzhou Institutes of Biomedicine and Health, Chinese Academy of Sciences, Guangzhou, China; ^2^ University of Chinese Academy of Sciences, Beijing, China; ^3^ Institute of Biological and Medical Engineering, Guangdong Academy of Sciences, Guangzhou, China; ^4^ National Engineering Research Center for Healthcare Devices, Guangzhou, China; ^5^ Department of Anatomy and Neurobiology, Xiangya School of Medicine, Central South University, Changsha, China

**Keywords:** gut microbiota dysbiosis, cognitive dysfunction, neuroinflammation, prenatal valproic acid exposure, autism spectrum disorder; ASD

## Abstract

**Introduction:**

Prenatal exposure to valproic acid (VPA) is a recognized risk factor for autism spectrum disorder (ASD)-like phenotypes, yet the mechanisms linking gut microbiota dysbiosis to neurodevelopmental impairments remain poorly understood. Emerging evidence implicates the microbiota-gut-brain axis as a critical mediator of neuroinflammation and cognitive deficits, but causal pathways in VPA-induced ASD models require systematic exploration. This study investigates how prenatal VPA exposure reshapes gut microbiota composition, exacerbates neuroinflammatory responses, and drives cognitive dysfunction through the microbiota-gut-brain axis in C57BL/6 mouse offspring.

**Methods:**

Prenatal VPA-exposed and control offspring underwent behavioral assessments (open field, three-chamber social interaction, marble-burying, and Morris water maze tests). Neuroinflammatory cytokines (IL-1β, IL-6, TNF-α, IL-10), oxidative stress markers (GSH, SOD, MDA), and microglial activation (Iba1 immunofluorescence) were quantified. Gut microbiota profiles were analyzed via 16S rRNA sequencing, with functional pathway predictions using PICRUSt2 and KEGG databases.

**Results:**

VPA-exposed mice exhibited ASD-like behaviors, including social deficits, repetitive stereotypic actions, and impaired spatial memory. Neuroinflammation was marked by upregulated pro-inflammatory cytokines (IL-1β, IL-6, TNF-α) and microglial hyperactivation, alongside suppressed antioxidant systems (GSH, SOD). Gut microbiota analysis revealed dysbiosis characterized by reduced Bacteroidia and enriched Clostridia, with diminished short-chain fatty acid (SCFA)-producing taxa (e.g., Oscillibacter). Co-occurrence networks highlighted disrupted microbial interactions, while functional profiling indicated impaired carbohydrate metabolism and elevated neurotoxic pathway activity.

**Discussion:**

Prenatal VPA exposure induces gut microbiota remodeling that exacerbates neuroinflammation and cognitive dysfunction via the microbiota-gut-brain axis. This study provides evidence for linkages between taxonomic and metabolic gut dysbiosis and ASD-like pathophysiology, underscoring the therapeutic potential of microbiota-targeted interventions for neurodevelopmental disorders.

## Highlights

- Mechanistic Link Between Gut Dysbiosis and Neuroinflammation: Establishes prenatal VPA-induced gut microbiota remodeling (*Bacteroidia*↓) may as a driver of neuroinflammatory cytokine surges (IL-1β, IL-6, TNF-α) and microglial hyperactivation (Iba1↑) in ASD-like mice.
- SCFA Depletion and Neurotoxic Pathway Activation: Identifies reduced SCFA-producing taxa (*Oscillibacter*↓) and elevated neurotoxic metabolic pathways may as key mediators of cognitive dysfunction.
- Integrated Multi-Omics Profiling: Combines 16S rRNA sequencing, co-occurrence networks, and KEGG functional analysis to decode microbial-immune-behavioral interactions.
- Microbiota-Driven Neuroimmune Dysregulation: Reveals gut dysbiosis may as a trigger for prefrontal/hippocampal microglial activation, linking microbial shifts to spatial memory deficits.
- Therapeutic Implications: Proposes microbiota modulation (e.g., SCFA restoration) may as a strategy to mitigate ASD-like phenotypes.

## 1 Introduction

The gut microbiota modulates neurodevelopment through immune, metabolic, and neural pathways via the microbiota-gut-brain axis (MGBA) (1, 2). Dysregulated MGBA signaling is implicated in neurological disorders including ASD, where neuroinflammation and cognitive dysfunction are hallmark features (3, 4). Prenatal exposure to environmental or pharmacological stressors, such as valproic acid (VPA), a commonly prescribed antiepileptic drug, is recognized as a significant risk factor for ASD-like phenotypes in offspring (5, 6). However, the mechanisms underlying how prenatal VPA exposure alters gut microbiota composition and subsequently impacts neurodevelopmental outcomes remain poorly understood.

Emerging evidence links maternal immune activation, oxidative stress, and gut microbiota dysbiosis to neurodevelopmental deficits in ASD models. Notably, prenatal VPA exposure alters gut microbiota composition, decreasing beneficial bacteria (Lactobacillus, Bifidobacterium) while increasing pro-inflammatory species (Enterobacteriaceae, Pseudomonadota). (5). These microbial shifts are associated with increased neuroinflammatory cytokines, such as interleukin-1β (IL-1β), interleukin-6 (IL-6), and tumor necrosis factor-alpha (TNF-α), which have been implicated in synaptic plasticity and cognitive deficits (7, 8). Furthermore, the gut microbiota’s role in producing short-chain fatty acids (SCFAs), critical for maintaining gut barrier integrity and modulating neuroimmune responses, has emerged as a key factor linking microbial dysbiosis to neurodevelopmental disorders (4, 9).

Key knowledge gaps persist regarding prenatal VPA exposure, gut microbiota changes, and neurodevelopment: (1) The causal mechanisms connecting microbial dysbiosis to neuroinflammation and cognitive deficits remain unclear; (2) The MGBA’s mediating role, particularly how microbial metabolites affect CNS function, is poorly understood; (3) Sex-specific effects of dysbiosis are understudied despite ASD’s male predominance. To address these limitations, the present study investigates how prenatal VPA exposure reshapes gut microbiota composition in C57BL/6 mouse offspring and examines the downstream effects on neuroinflammation and cognitive dysfunction via the MGBA. By 16S rRNA sequencing, this study provides a comprehensive analysis of microbial community structure and functional pathways associated with VPA-induced ASD-like phenotypes.

Our hypothesis is that prenatal VPA exposure induces gut microbiota dysbiosis, which exacerbates neuroinflammation and cognitive dysfunction in offspring through the MGBA. Specifically, we propose that: ① Prenatal VPA exposure alters gut microbiota composition, characterized by reduced SCFA-producing taxa and enrichment of pro-inflammatory and neurotoxic bacterial lineages. ② These microbial shifts are associated with increased neuroinflammatory cytokines and oxidative stress markers in the CNS.

Unlike previous studies that have focused on isolated aspects of this relationship, our approach integrates comprehensive microbial profiling with functional analyses of neuroinflammatory and cognitive endpoints. Additionally, our use of a well-established C57BL/6 mouse model of prenatal VPA exposure ensures robust translational relevance to human ASD. By identifying specific microbial taxa and pathways implicated in neurodevelopmental impairments, this study provides a foundation for developing novel therapeutic strategies targeting the gut microbiota as a modifiable factor in neurodevelopmental disorders.

This study elucidates how prenatal VPA exposure affects neurodevelopment through gut microbiota changes. Our findings advance MGBA understanding and provide preclinical support for microbiota-targeted ASD interventions, particularly in males.

## 2 Materials and methods

### 2.1 Ethics statement

All experimental protocols were approved by the Institutional Animal Care and Use Committee (IACUC) of the Guangzhou Institute of Biomedicine and Health, Chinese Academy of Sciences (Approval No. CAS [IACUC:2023081]) and conducted in accordance with the Guide for the Care and Use of Laboratory Animals (National Research Council, 8th edition, 2011). Procedures adhered to the 2020 AVMA Guidelines for Euthanasia and the ARRIVE 2.0 reporting guidelines (Percie du Sert et al., 2020). All efforts were made to minimize animal suffering, including the use of humane endpoints and appropriate analgesic regimens.

#### 2.1.1 Animals and experimental design

##### 2.1.1.1 Animals

Adult C57BL/6 mice (18 females, 9 males; 20–25 g initial body weight) were housed under specific pathogen-free (SPF) conditions in a temperature-controlled environment (22 ± 1°C, 55 ± 5% humidity) with a 12-hour light/dark cycle (lights on: 07:00). Animals received autoclaved water and standard rodent chow (Guangdong Medical Laboratory Animal Center) ad libitum.

##### 2.1.1.2 Breeding protocol

Male and female mice were co-housed at 17:00, and vaginal plugs were checked at 09:00 the following morning. Plug-positive females were designated as gestational day (GD) 0.5.

##### 2.1.1.3 Prenatal VPA exposure

Valproic acid (VPA)-induced rodent models are widely utilized to study autism spectrum disorder (ASD) but suffer from high maternal mortality and inconsistent phenotypic outcomes due to imprecise dosing timing and acute embryotoxicity. To overcome limitations of traditional single-dose valproic acid (VPA) models in autism spectrum disorder (ASD) research—including severe maternal toxicity and imprecise embryonic exposure—we have developed a triple-phase dosing strategy (patent no. P24GZ1NNO9183CN), which optimizes embryonic exposure timing and phenotypic consistency.

This study follows the previously established three-stage patentable drug administration induction method, and is described briefly as follows: Twenty-four pregnant dams were stratified-randomized into two groups:

VPA group (n = 12 dams): Received daily intraperitoneal (i.p.) injections of sodium valproate (VPA; Sigma-Aldrich, #P4543) dissolved in endotoxin-free saline at 300 mg/kg (GD11.5), 400 mg/kg (GD12.5), and 300 mg/kg (GD13.5) to target peak neocortical neurogenesis.

Control: The control group received saline injections (600 mg/kg) at all three gestational time points (E11.5, E12.5, and E13.5) to match the experimental protocol and control for potential effects of repeated injections.

The dose selection for modified regimens was based on: (i) literature-reported effective thresholds for ASD phenotyping (10); and (ii) pilot dose-ranging studies in our lab balancing embryotoxicity avoidance and behavioral phenotype induction.

##### 2.1.1.4 Postnatal allocation

Male offspring were cross-fostered at birth to eliminate litter effects. At postnatal day 21 (PND21), pups were randomly assigned to:

Control group (n = 12): Saline-treated offspring from control dams.

VPA group (n = 12): VPA-exposed offspring.

### 2.2 Behavioral tests

All behavioral assessments were conducted in a dedicated testing room under controlled conditions (23 ± 1°C, 50 lux illumination). Mice were habituated to the testing environment for 1 hour prior to each procedure. Experimenters were blinded to group assignments throughout data collection and analysis.

#### 2.2.1 Three-chamber social interaction test

**Apparatus:**

A rectangular polycarbonate arena (90 × 30 × 25 cm) divided into three equal compartments by retractable doors.

**Habituation Protocol:**

Days 1–7: Daily 2-hour exposure to testing conditions.

Days 8–10: Gradual acclimatization:

Central compartment exploration: 3 min/day.

Full arena access: 3 min/day.

**Experimental Phases:**

Baseline Phase (5 min): Both lateral chambers contained empty wire cages.

**Sociability Test (0–10 min):**

Social stimulus: Age-/sex-matched C57BL/6 mouse (Stranger 1) in one cage.

Control: Empty cage in the opposite chamber.

**Social Novelty Preference (10–20 min):**

Novel stimulus: Stranger 2 (novel C57BL/6 mouse) replaced the control cage.

Familiar stimulus: Stranger 1 remained in the original position.

**Key Controls:**

Stranger mice: Individually housed for 48 hours pre-test to standardize olfactory cues.

Tracking: Interaction time (snout <2 cm from cage) quantified using EthoVision XT 15.0 (Noldus IT, Netherlands).

#### 2.2.2 Self-grooming test

Apparatus: Clean polycarbonate cages under 50 lux illumination.

**Protocol:**

Habituation: 10 min free exploration.

Testing: 10 min video recording (Logitech C920 HD Pro) of spontaneous grooming behaviors, including: Facial cleaning, Body licking, Genital/tail grooming.

Analysis:

Bout frequency: Number of discrete grooming episodes.

Cumulative duration: Total time engaged in stereotypic movements.

Three blinded observers analyzed recordings; inter-rater reliability >95% (Cohen’s κ).

#### 2.2.3 Marble-burying test

Apparatus: Polycarbonate chambers with 5 cm corncob bedding (Harlan Laboratories).

Protocol:

Acclimatization: 3 min free exploration.

Testing: 16 black glass marbles (1.6 mm diameter) arranged in a 4×4 grid (3 cm spacing).

Session: 10 min video recording under 50 lux.

Scoring Criteria:

Burial definition: ≥75% marble surface covered by bedding.

Consensus: ≥2/3 observers agreement. Ambiguous cases resolved via frame-by-frame analysis.

#### 2.2.4 Open field test

Apparatus: Square arena (50 × 50 × 30 cm) with a central zone (30 × 30 cm).

Protocol:

Habituation: 10 min free exploration.

Testing: 10 min recording using EthoVision XT 15.0 (Noldus IT).

Parameters:

Central/peripheral crossings (all limbs crossing zone borders).

Vertical activity (forelimbs raised ≥2 s).

#### 2.2.5 Morris water maze

Apparatus:

Black circular pool (120 cm diameter) filled with opacified water (23.0 ± 0.5°C; nontoxic white tempera).

Hidden platform (10 cm diameter, 1.5 cm submerged).

Spatial cues: Four high-contrast geometric patterns (50 ± 5 lux uniformity).

Protocol:

**Acquisition Phase (5 days):**

4 trials/day (90 s max, 25 min inter-trial interval).

Entry points randomized (N, S, E, W).

Platform training: 10 s enforced orientation for failed trials.

**Probe Trial:**

Conducted 24 h post-training (platform removed).

60 s free swim to assess spatial memory retention.

Tracking: EthoVision XT 15.0 with <2% positional error (chessboard-calibrated).

### 2.3 Tissue collection and histological processing

Following behavioral assessments, mice were deeply anesthetized via intraperitoneal injection of ketamine/xylazine (100/10 mg/kg) and transcardially perfused with ice-cold 0.9% NaCl followed by 4% paraformaldehyde (PFA; Merck, Germany). Brains were post-fixed in 4% PFA for 24 hr at 4°C, after which the hippocampus and prefrontal cortex were dissected. Tissues were dehydrated through graded ethanol series, embedded in paraffin (Merck), and sectioned coronally at 5 μm thickness using a rotary microtome (Microm HM335E, Walldorf, Germany). Sections were mounted on Superfrost Plus slides (Thermo Fisher) for subsequent analyses.

### 2.4 Biochemical and cytokine profiling

Oxidative stress markers (GSH, T-SOD, GSH-PX, MDA, CAT) and nitric oxide metabolites (T-NOS, NO) were quantified using commercial kits (Nanjing Jiancheng Institute of Biotechnology) following manufacturer protocols. Pro- and anti-inflammatory cytokines (IL-6, IL-1β, TNF-α, IL-10) were measured in serum using mouse-specific ELISA kits (Fankel, Shanghai) with inter- and intra-assay coefficients of variation <10%. Absorbance was read on a SpectraMax M5 microplate reader (Molecular Devices).

### 2.5 Gut microbiota 16S rRNA sequencing

Sample Collection: Fresh fecal pellets (3–5 pellets/mouse, ~100 mg) were collected aseptically between 09:00–11:00 to minimize circadian variability. Samples were snap-frozen in liquid nitrogen within 5 min and stored at −80°C until processing.

#### 2.5.1 DNA extraction and sequencing

Genomic DNA was extracted using a modified CTAB protocol with mechanical homogenization (0.1 mm zirconium beads, FastPrep-24 homogenizer). DNA purity (A260/A280 = 1.8–1.9, A260/A230 > 2.0) was verified via NanoDrop 2000. The V3–V4 hypervariable regions were amplified with barcoded primers 341F/806R using Phusion^®^ High-Fidelity PCR Master Mix (New England Biolabs). Libraries were constructed with the NEBNext Ultra II DNA Library Prep Kit (Illumina) and sequenced on an Illumina NovaSeq 6000 platform (PE250 mode, 50,000 reads/sample).

#### 2.5.2 Bioinformatics analysis

Raw sequences were processed in QIIME2 (v2023.2) using DADA2 for denoising and chimera removal. Taxonomic classification was performed against the SILVA 138.1 database. α-diversity (Chao1, Shannon), β-diversity (Bray-Curtis, UniFrac), and differential taxa were analyzed via LEfSe (LDA >2.0), ANCOM (W >0.7), and DESeq2 (FDR <0.05). Functional pathways were predicted using PICRUSt2 (KEGG database) and visualized in STAMP. Co-occurrence networks (Spearman |ρ| >0.6, p < 0.01) were constructed with Gephi (v0.10.1). PICRUSt2-predicted pathways represent inferred functional potential; metagenomic/metabolomic validation is needed for confirmation.

### 2.6 Immunofluorescence staining

Brain sections underwent standard deparaffinization and antigen retrieval protocols. After three washes (5 min each) with phosphate-buffered saline (PBS, pH 7.4), nonspecific binding was blocked by incubating sections with 4% (w/v) bovine serum albumin (BSA) in PBS containing 0.3% Triton X-100 (PBST) for 1 h at room temperature (RT). Sections were then incubated with rabbit primary antibodies (1:200 dilution in blocking solution) targeting specific antigens at 4°C for 16 h under humidified conditions. Following PBST washes (3 × 10 min), samples were incubated with Alexa Fluor 488-conjugated goat anti-rabbit IgG secondary antibodies in the dark for 1 h at RT. Nuclei were counterstained with 4′,6-diamidino-2-phenylindole (DAPI; 1 μg/mL) for 10 min in the dark.

High-resolution z-stack images were captured using a confocal laser scanning microscope (TCS SP8 STED 3X, Leica Microsystems) with standardized laser power and detector gain settings. Integrated density (IntDen) values were quantified using ImageJ software (National Institutes of Health, Bethesda, MD, USA).

### 2.7 Statistical analysis

Statistical analyses were performed using GraphPad Prism software. Continuous data are presented throughout the figures and text as mean ±SD. Normality of data distribution was assessed using the Shapiro-Wilk test. For comparisons between the two experimental groups, an unpaired two-tailed Student's t -test was employed when data met assumptions of normality and homogeneity of variance. Where data violated normality assumptions, the non-parametric Mann-Whitney U test was used instead. Statistical significance was defined as a p -value less than 0.05 for all analyses.

## 3 Results

### 3.1 Open field test behavioral analysis

**Figure 1:**
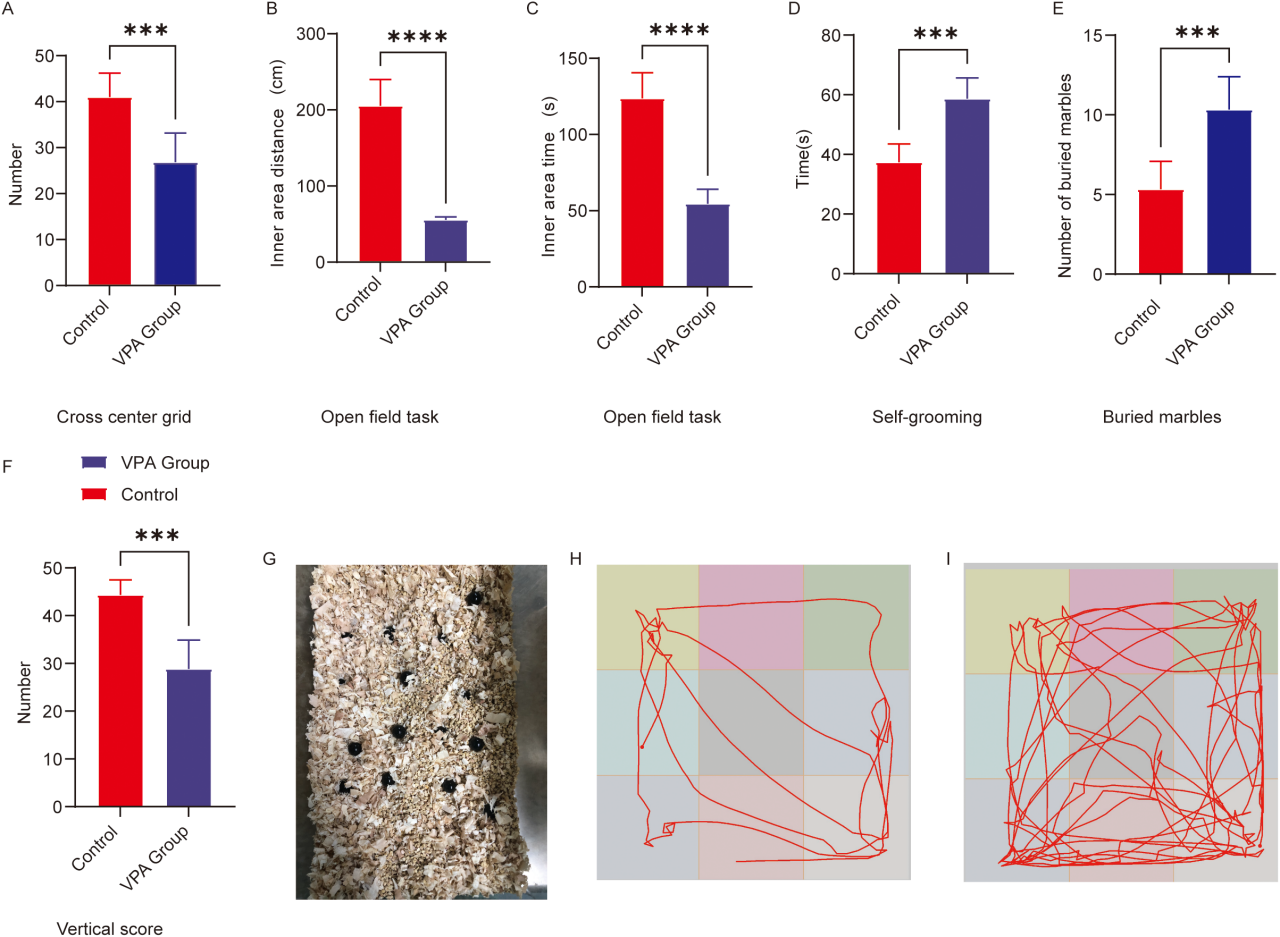
Behavioral analysis of C57BL/6 mice in the open field and repetitive stereotypic behaviors. **(A)** Cross-center grid; **(B)** inner area distance; **(C)** inner area time; **(D)** self-grooming; **(E)** buried marbles; **(F)** vertical score; **(G)** Representative diagram of buried marbles; **(H)** Representative open field trajectory diagram of the ASD model; **(I)** Representative open field trajectory map of the control group. The data are presented as the mean ± SD (x̅ ± s, n=12). Statistical significance: ***p < 0.001, ****p < 0.0001, compared with the control group.

The open field test was conducted to evaluate autonomous exploratory behaviors and anxiety-like responses in C57BL/6 mice exposed to a novel environment. Valproic acid (VPA) exposure induced significant reductions in exploratory activity and exacerbated anxiety-like behaviors compared to control mice (**Figure 1**). Key findings are detailed below:

#### 3.1.1 Exploratory behaviors

Center grid crossings (**Figure 1A**): VPA-exposed mice exhibited a marked reduction in center grid crossings relative to controls (P < 0.001).

Inner zone movement distance (**Figure 1B**): The total distance traveled within the inner zone was significantly shorter in the VPA-treated group compared to controls (P < 0.0001).

#### 3.1.2 Enhanced anxiety-like behaviors

**Inner area time** (**Figure 1C**): Activity time was significantly shorter in the group of C57BL/6 mice exposed to VPA than in the control group (P<0.0001). **Vertical score** (**Figure 1F**): Scores in the group of C57BL/6 mice exposed to VPA were significantly lower than those in the control group (P < 0.001).

Representative locomotor trajectories further illustrated these behavioral differences: VPA-exposed mice displayed restricted movement patterns (**Figure 1H**), whereas control mice explored the arena more extensively (**Figure 1I**).

### 3.2 Analysis of repetitive stereotypic behavior

#### 3.2.1 Buried marble test

The marble-burying test, a classical paradigm for assessing repetitive stereotypic behaviors in rodents, revealed that valproic acid (VPA) exposure significantly enhanced marble-burying behavior in C57BL/6 mice (**Figure 1E**). VPA-exposed mice buried significantly more marbles compared to controls (P < 0.001; **Figure 1E**). A representative diagram of buried marbles is shown in **Figure 1G**.

#### 3.2.2 Self-grooming

VPA-exposed mice exhibited excessive self-grooming behavior (**Figure 1D**). Grooming frequencies were significantly higher in the VPA-treated group than in controls (P < 0.001).

Conclusion:

C57BL/6 mice exposed to VPA displayed increased repetitive stereotypic behaviors, including marble-burying and self-grooming, suggesting neurodevelopmental anomalies potentially linked to basal ganglia-cortical circuit dysfunction.

### 3.3 Three-chamber social behavioral analysis

The three-chamber test evaluated social motivation (0–10 min) and social novelty preference (10–20 min) in C57BL/6 mice by quantifying exploration time toward unfamiliar conspecifics (Stranger 1/2) versus empty cages/objects.

**I. Social Motivation Phase (Sociability, 0–10 min)**

**Figure 2:**
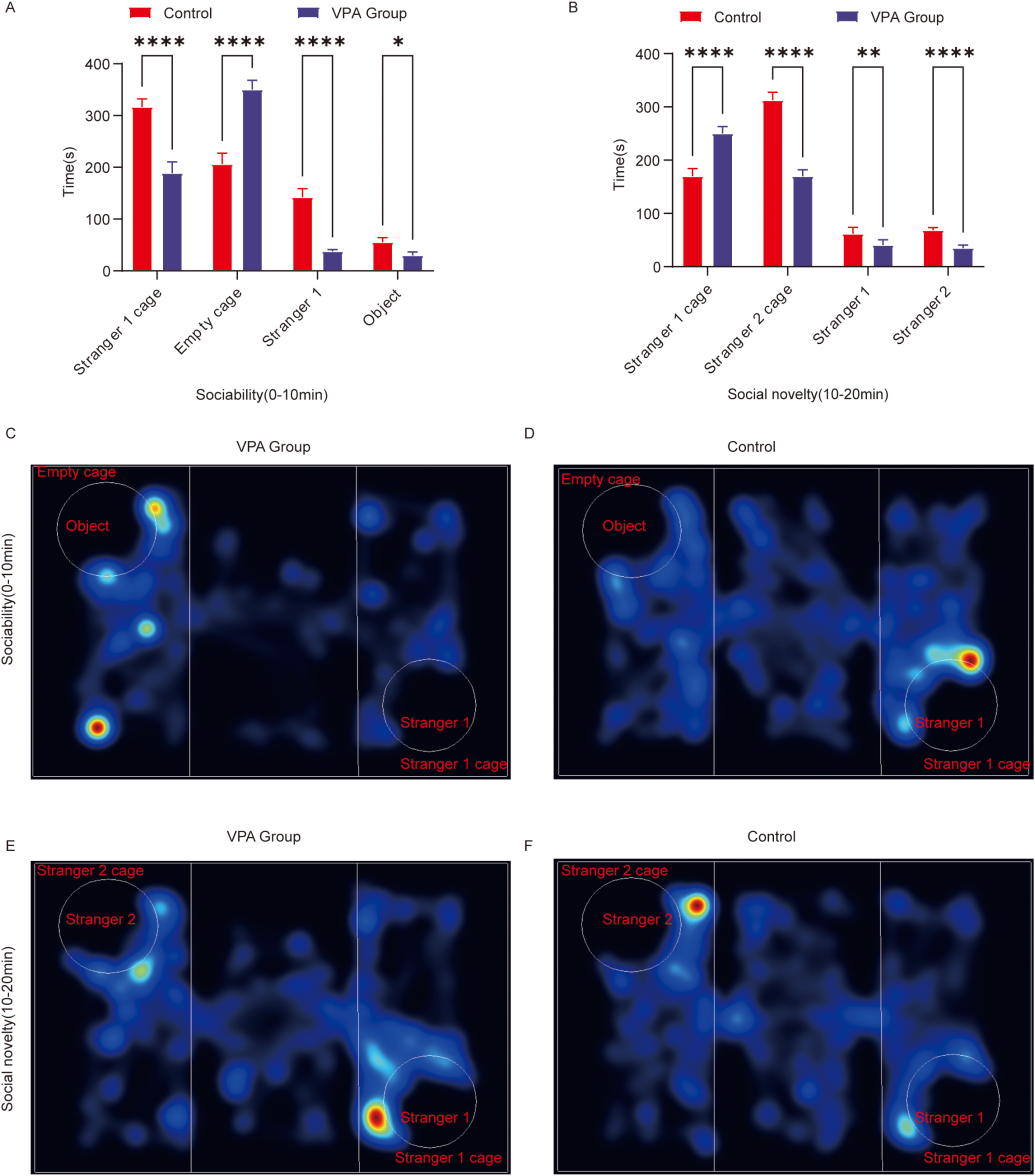
Behavioral analysis of three-chamber social behavior in C57BL/6 mice. **(A)** Social ability; **(B)** social novelty; **(C)** Representative heat map of social ability (0–10 min) in the ASD model group; **(D)** Representative heat map of social ability (0–10 min) in the control group; **(E)** Representative heat map of the social novelty preference phase (10–20 minutes) in the ASD model group; **(F)** Representative heat map of the social novelty preference phase (10–20 minutes) in the control group. The data are presented as the mean ± SD (x̅ ± s, n=12). Statistical significance: *p < 0.05, **p < 0.01, ****p < 0.0001, compared with the control group. Stranger 1 cage: time spent in the stranger 1 cage; Empty cage: time spent in the object cage; stranger 1: interaction time with stranger 1; object: interaction time with the object; stranger 2 cage: time spent in the stranger 2 cage; stranger 2: interaction time with stranger 2.

VPA-exposed mice exhibited markedly reduced social motivation compared to controls (**Figure 2A**):

**(1) Spatial Exploration Preference**

Stranger 1 cage duration: VPA-exposed mice spent significantly less time in the Stranger 1 cage than controls (P < 0.0001).

Empty cage duration: VPA-exposed mice showed a pronounced preference for the empty cage over the Stranger 1 cage (P < 0.0001).

**(2) Social Interaction Behavior**

Stranger 1 sniffing time: Interaction durations with Stranger 1 were significantly shorter in VPA-exposed mice than in controls (P < 0.0001).

Object sniffing time: VPA-exposed mice also displayed reduced interaction times with objects compared to controls (P < 0.05).

Representative locomotor trajectories during the sociability phase are shown for the ASD model group (**Figure 2C**) and control group (**Figure 2D**).

Conclusion: VPA-exposed mice avoided interactions with unfamiliar conspecifics (Stranger 1) and preferentially engaged with nonsocial contexts (empty cage), indicating impaired social competence (P < 0.0001).

**II. Social Novelty Preference Phase (10–20 min;** **Figure 2B**)

**(1) Spatial Exploration Patterns**

Stranger 1 cage duration: VPA-exposed mice spent significantly more time in the Stranger 1 cage than controls (P < 0.0001).

Stranger 2 cage duration: Interaction time with the novel Stranger 2 cage was significantly reduced in VPA-exposed mice compared to controls (P < 0.0001).

**(2) Social Interaction Disparities**

Stranger 1 sniffing time: VPA-exposed mice exhibited increased interaction with Stranger 1 compared to controls (P < 0.01).

Stranger 2 sniffing time: Exploration of the novel social stimulus (Stranger 2) was significantly diminished in VPA-exposed mice (P < 0.0001).

Representative trajectories for the novelty preference phase are shown for the ASD model group (**Figure 2E**) and control group (**Figure 2F**).

Conclusion: VPA-exposed mice displayed restricted interest in social novelty and failed to exhibit a clear preference for novel stimuli.

### 3.4 Assessment of learning and memory capacity

The Morris water maze (MWM) was employed to evaluate spatial learning and memory in C57BL/6 mice through two phases: spatial acquisition trials and spatial probe trials.

#### 3.4.1 Spatial acquisition trials

**Figure 3:**
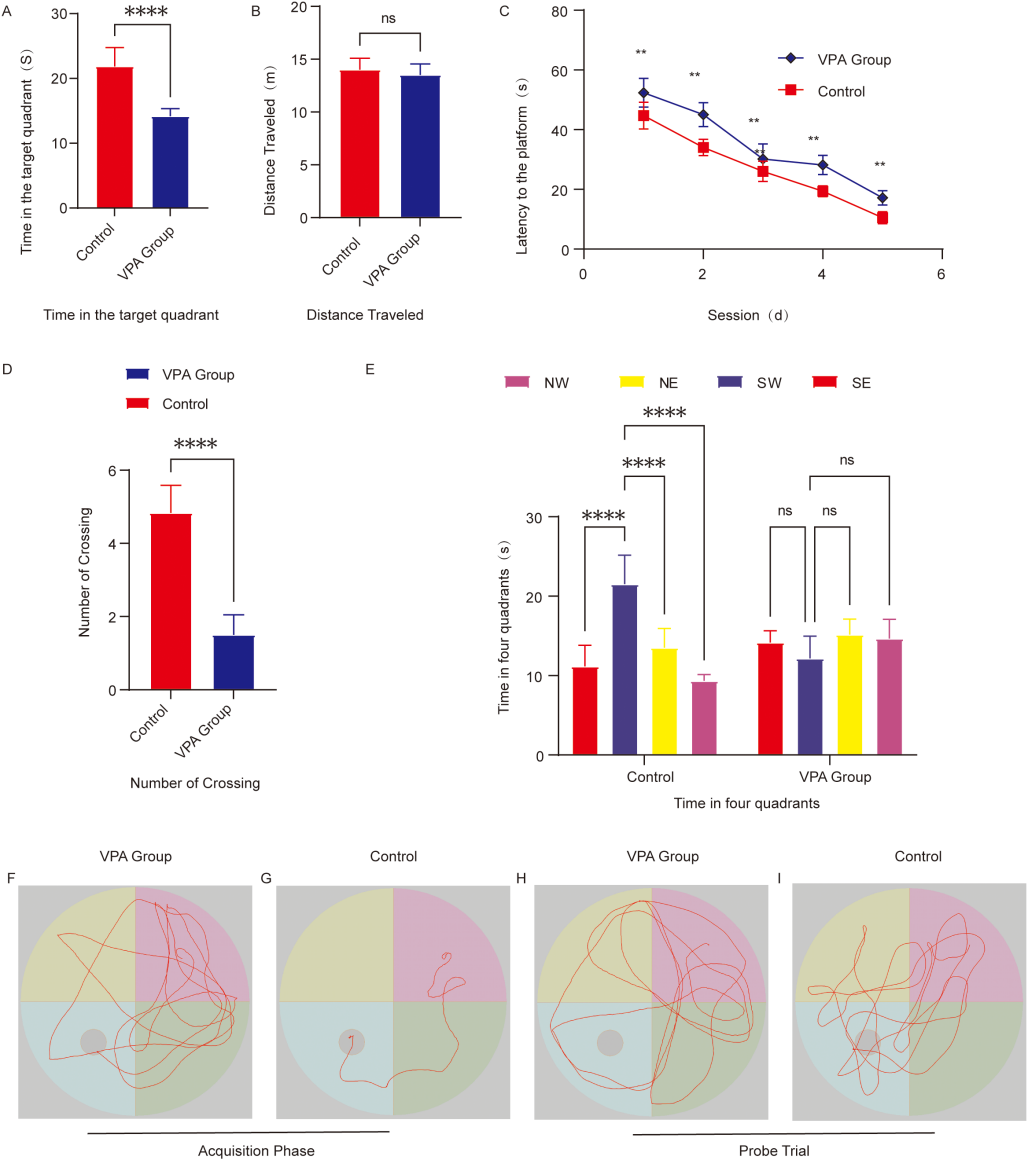
Behavioral analysis of the Morris water maze. **(A)** Time spent in the target quadrant. **(B)** Total swimming distance. **(C)** Escape latency to locate the hidden platform. **(D)** Number of platform crossings. **(E)** Time distribution across the four quadrants during the spatial probe trial. **(F)** Representative swimming paths of the group of C57BL/6 mice exposed to VPA during spatial acquisition trials. **(G)** Representative Swimming paths of control mice during spatial acquisition trials. **(H)** Representative Exploration trajectories of the group of C57BL/6 mice exposed to VPA during spatial probe trials. **(I)** Representative Exploration trajectories of control mice during spatial probe trials. The data are presented as the mean ± SD (x̅ ± s, n=12). Statistical significance: **p < 0.01, ****p < 0.0001, compared with the control group.

During training, VPA-exposed mice displayed disorganized, random search patterns to locate the hidden platform (**Figure 3F**), whereas control mice adopted goal-directed navigation strategies, with some individuals swimming directly to the platform using spatial memory (**Figure 3G**). VPA-exposed mice exhibited significantly prolonged escape latencies compared to controls (P < 0.01; **Figure 3C**).

#### 3.4.2 Spatial probe trials

Quadrant residence time: VPA-exposed mice showed no target quadrant preference (P > 0.05), while controls spent significantly more time in the target quadrant than in other quadrants (P < 0.0001; **Figure 3E**).

Platform crossings: The number of platform crossings was significantly reduced in VPA-exposed mice compared to controls (P < 0.0001; **Figure 3D**).

Target quadrant duration: VPA-exposed mice spent markedly less time in the target quadrant than controls (P < 0.0001; **Figure 3A**), indicating impaired spatial memory retention.

Swimming distance: No significant intergroup differences in total swimming distance were observed (P > 0.05; **Figure 3B**), confirming intact motor function in VPA-exposed mice.

Representative swimming trajectories revealed that control mice focused exploration on the original platform quadrant, frequently crossing the target area (**Figure 3I**), whereas VPA-exposed mice engaged in aimless exploration across all quadrants (**Figure 3H**).

Conclusion:

VPA-exposed mice displayed significant deficits in spatial learning and memory, characterized by prolonged escape latencies, random search strategies, and reduced target quadrant preference. These cognitive impairments occurred independently of locomotor dysfunction, highlighting the specificity of neurobehavioral deficits in VPA-exposed mice.

### 3.5 Analysis of neuroinflammatory levels in the prefrontal cortex

**Figure 4:**
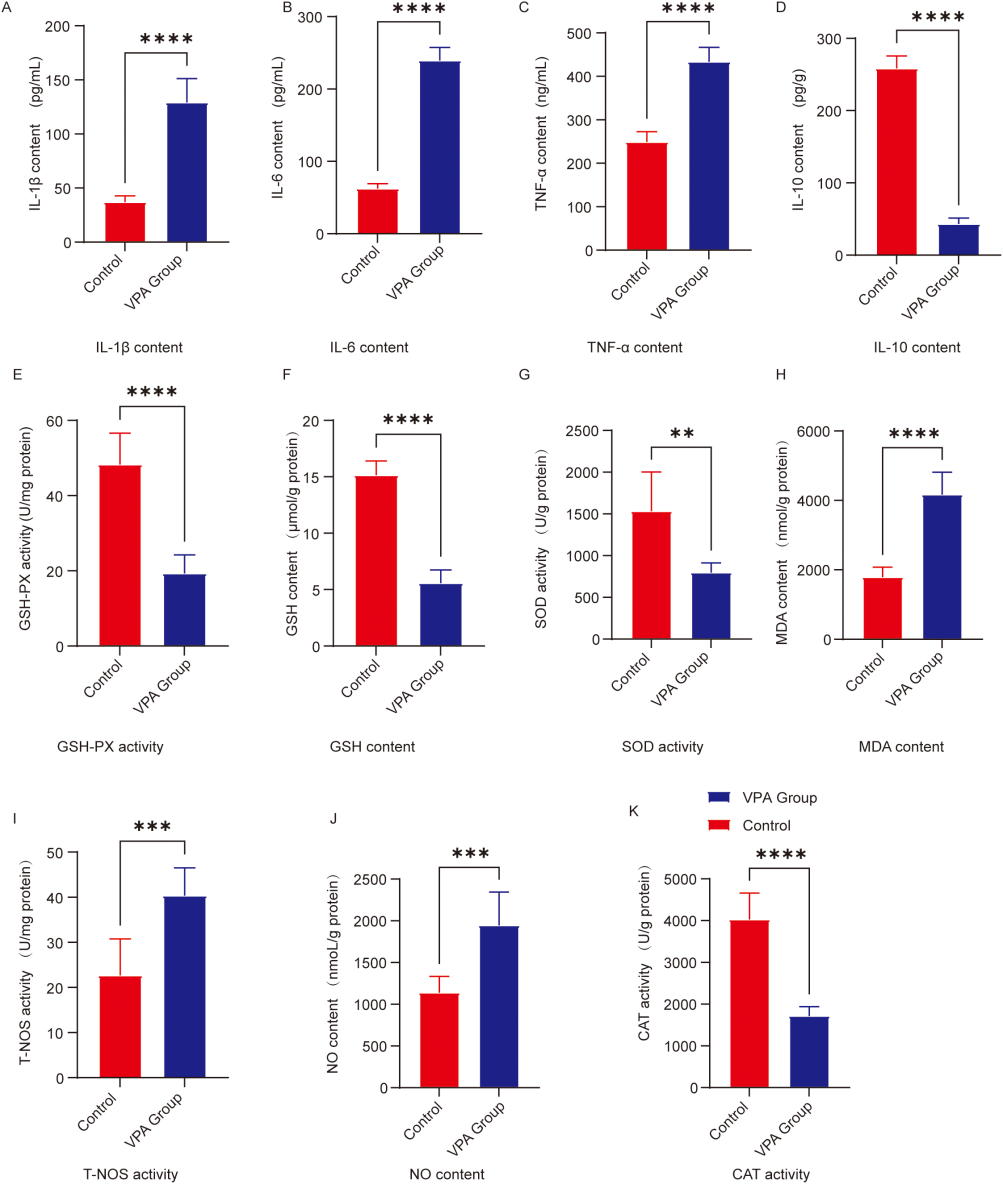
Analysis of neuroinflammatory and oxidative stress levels in the prefrontal cortex. **(A)** IL-1β **(B)** IL-6 **(C)** TNF-α **(D)** IL-10 **(E)** GSH-Px activity; **(F)** GSH content; **(G)** SOD activity; **(H)** MDA content; **(I)** T-NOS activity; **(J)** NO content; **(K)** CAT activity. The data are presented as the mean ± SD (x̅ ± s, n=12). Statistical significance: **p < 0.01, ***p < 0.001, ****p < 0.0001, compared with the control group.

ELISAs demonstrated that valproic acid (VPA) exposure significantly altered the inflammatory cytokine profile in the prefrontal cortex of C57BL/6 mice (**Figure 4**). Key results are summarized below:

#### 3.5.1 Proinflammatory cytokine upregulation

IL-1β (**Figure 4A**): IL-1β levels were significantly elevated in VPA-exposed mice compared to controls (P < 0.0001).

IL-6 (**Figure 4B**): IL-6 levels showed a marked increase in VPA-exposed mice versus controls (P < 0.0001).

TNF-α (**Figure 4C**): TNF-α content was significantly higher in VPA-exposed mice than in controls (P < 0.0001).

#### 3.5.2 Anti-inflammatory cytokine downregulation

IL-10 (**Figure 4D**): IL-10 levels were significantly reduced in VPA-exposed mice relative to controls (P < 0.0001).

Conclusion: VPA exposure disrupted the proinflammatory/anti-inflammatory balance in the prefrontal cortex of mice.

### 3.6 Analysis of oxidative stress levels in the prefrontal cortex

ELISAs revealed that valproic acid (VPA) exposure significantly disrupted oxidative stress homeostasis in the prefrontal cortex of C57BL/6 mice (**Figure 4**). Specific findings are detailed below:

#### 3.6.1 Suppression of antioxidant systems

Catalase (CAT; **Figure 4K**): CAT levels were significantly reduced in VPA-exposed mice compared to controls (P < 0.0001).

Glutathione peroxidase (GSH-Px) activity (**Figure 4E**): GSH-Px activity decreased significantly in VPA-exposed mice (P < 0.0001).

Glutathione (GSH; **Figure 4F**): GSH content was markedly lower in VPA-exposed mice than in controls (P < 0.0001).

Superoxide dismutase (SOD; **Figure 4G**): SOD levels were significantly reduced in VPA-exposed mice versus controls (P < 0.01).

#### 3.6.2 Elevation of oxidative damage markers

Malondialdehyde (MDA; **Figure 4H**): MDA content was significantly higher in VPA-exposed mice than in controls (P < 0.0001).

Nitric oxide synthase (NOS) activity (**Figure 4I**): Total NOS (T-NOS) activity was substantially elevated in VPA-exposed mice compared to controls (P < 0.001).

Nitric oxide (NO) content (**Figure 4J**): NO levels were significantly increased in VPA-exposed mice relative to controls (P < 0.001).

Conclusion: VPA exposure induced a comprehensive decline in antioxidant capacity and significant accumulation of oxidative damage markers in the prefrontal cortex.

### 3.7 Microglial activation and neuroinflammation in ASD

To evaluate neuroinflammatory pathology in VPA-exposed C57BL/6 mice, immunofluorescence staining was performed to assess microglial activation via the marker Iba1.

**Figure 5:**
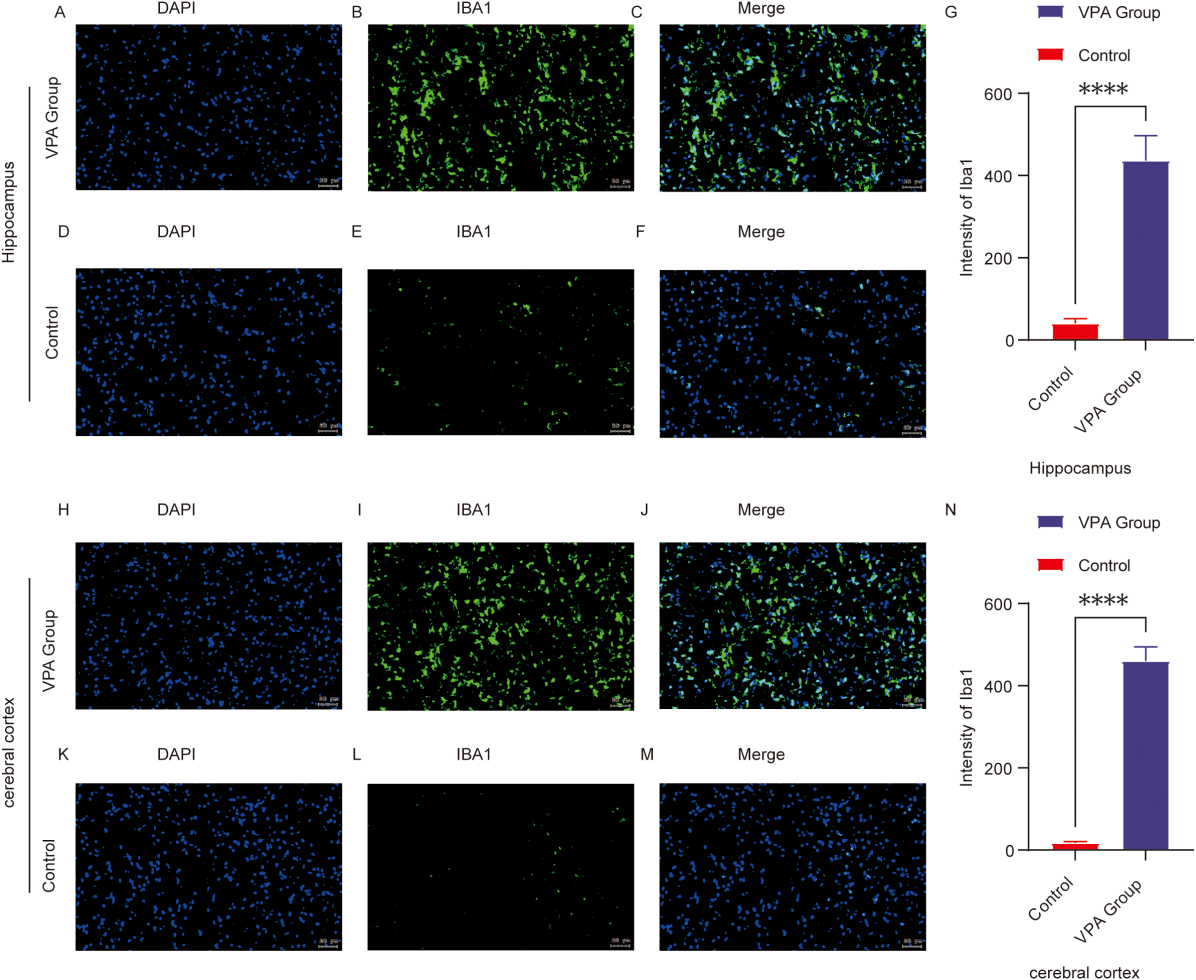
Immunofluorescence of Iba1 in the prefrontal cortex (PFC) and hippocampal CA1 region of control and ASD model mice. **(A–F)** Representative immunofluorescence images of Iba1 expression in the hippocampal CA1 region in the control **(D–F)** and VPA-exposed **(A–C)** groups. **(G)** Mean fluorescence intensity of Iba1 in the hippocampal CA1 region, expressed in arbitrary units. **(H–M)** Representative Iba1 immunofluorescence in the prefrontal cortex of the control **(K–M)** and VPA-exposed **(H–J)** groups. **(N)** shows the mean fluorescence intensity of Iba1 in the cerebral cortex, also expressed in arbitrary units. The data are expressed as arbitrary units (AUs) and were analyzed via ImageJ. The values represent the mean ± SD (****p < 0.0001, VPA *vs*. control; n = 6/group). For all the immunofluorescence images, Iba1 is stained green, and DAPI (nuclei) is stained blue. The scale bar in all the images represents 50 µm.

In the hippocampal CA1 region, Iba1 fluorescence intensity was significantly higher in VPA-exposed mice than in controls (P < 0.0001; **Figures 5A–G**). Similarly, in the prefrontal cortex, Iba1 fluorescence intensity was markedly elevated in VPA-exposed mice compared to controls (P < 0.0001; **Figures 5H–N**). These findings demonstrate that VPA exposure significantly increased microglial Iba1 expression in both the hippocampal CA1 region and prefrontal cortex relative to normal controls.

Conclusion: VPA exposure triggered microglial activation, shifting microglia from a resting state to a proinflammatory phenotype. This suggests neuroinflammatory mechanisms may contribute to ASD-related behavioral abnormalities.

### 3.8 Taxonomic and Compositional shifts in gut microbiota

**Figure 6:**
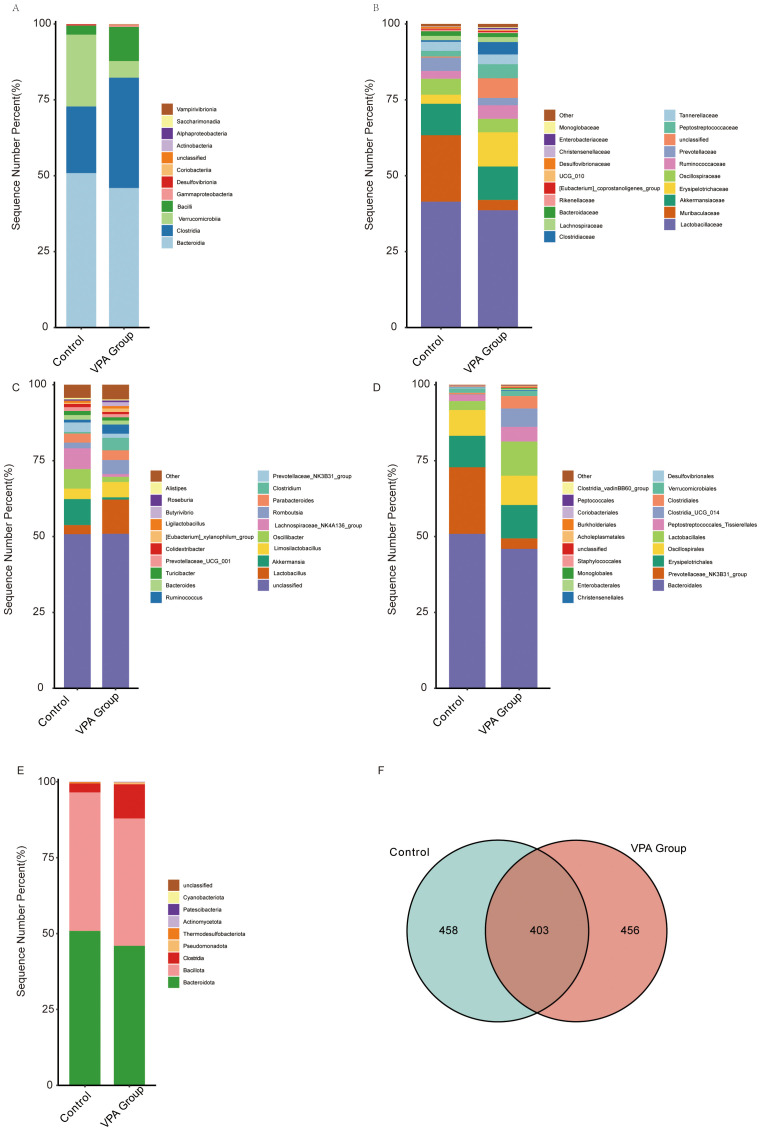
Gut microbiota compositional differences across taxonomic hierarchies in control and VPA-induced ASD mouse models. **(A)** Class_mean_barplot. **(B)** Family_mean_barplot. **(C)** Genus_mean_barplot. **(D)** Order_mean_barplot. **(E)** Phylum_mean_barplot. **(F)** Venn diagram.

Comparative analysis of gut microbiota across taxonomic hierarchies revealed distinct compositional differences between VPA-exposed mice and controls (**Figures 6A–F**).

Class level (**Figure 6A**): Clostridiales was enriched in the VPA group, suggesting dysbiosis favoring taxa linked to gut-brain axis modulation.

Family level (**Figure 6B**): Muribaculaceae (associated with mucosal homeostasis) was reduced in the VPA group.

Genus level (**Figure 6C**): *Oscillibacter* (butyrate-producing genus) was diminished in the VPA group.

Order level (**Figure 6D**): *Prevotellaceae_NK3B31_group* (linked to carbohydrate metabolism and short-chain fatty acid production) was reduced in the VPA group.

Phylum level (**Figure 6E**): *Bacteroidia* (critical for polysaccharide degradation) decreased in the VPA group, while Clostridia (pro-inflammatory taxa) increased.

Venn diagram (**Figure 6F**): Substantial overlap in operational taxonomic units (OTUs) between groups was observed, alongside unique OTU profiles indicating conserved and divergent microbial niches.

These taxonomic shifts reflect gut microbiota dysbiosis in the VPA-induced ASD model. Reduced *Bacteroidia* suggest altered metabolic and inflammatory pathways, while diminished *Oscillibacter* and *Muribaculaceae* may indicate impaired butyrate synthesis and mucosal barrier function. Conversely, the control group increased *Prevotellaceae_NK3B31_group* suggest adaptive microbial shifts potentially influencing neuroactive metabolite production.

The depletion of *Bacteroidia*, essential for dietary fiber fermentation, may compromise gut barrier integrity and anti-inflammatory metabolite synthesis. Reduced *Oscillibacter* (a butyrate producer) might disrupt gut-brain axis energy homeostasis. These findings highlight the gut microbiota as a dynamic interface in ASD pathophysiology, warranting further investigation into its role in neurobehavioral outcomes.

### 3.9 Taxonomic alterations in gut microbiota

**Figure 7:**
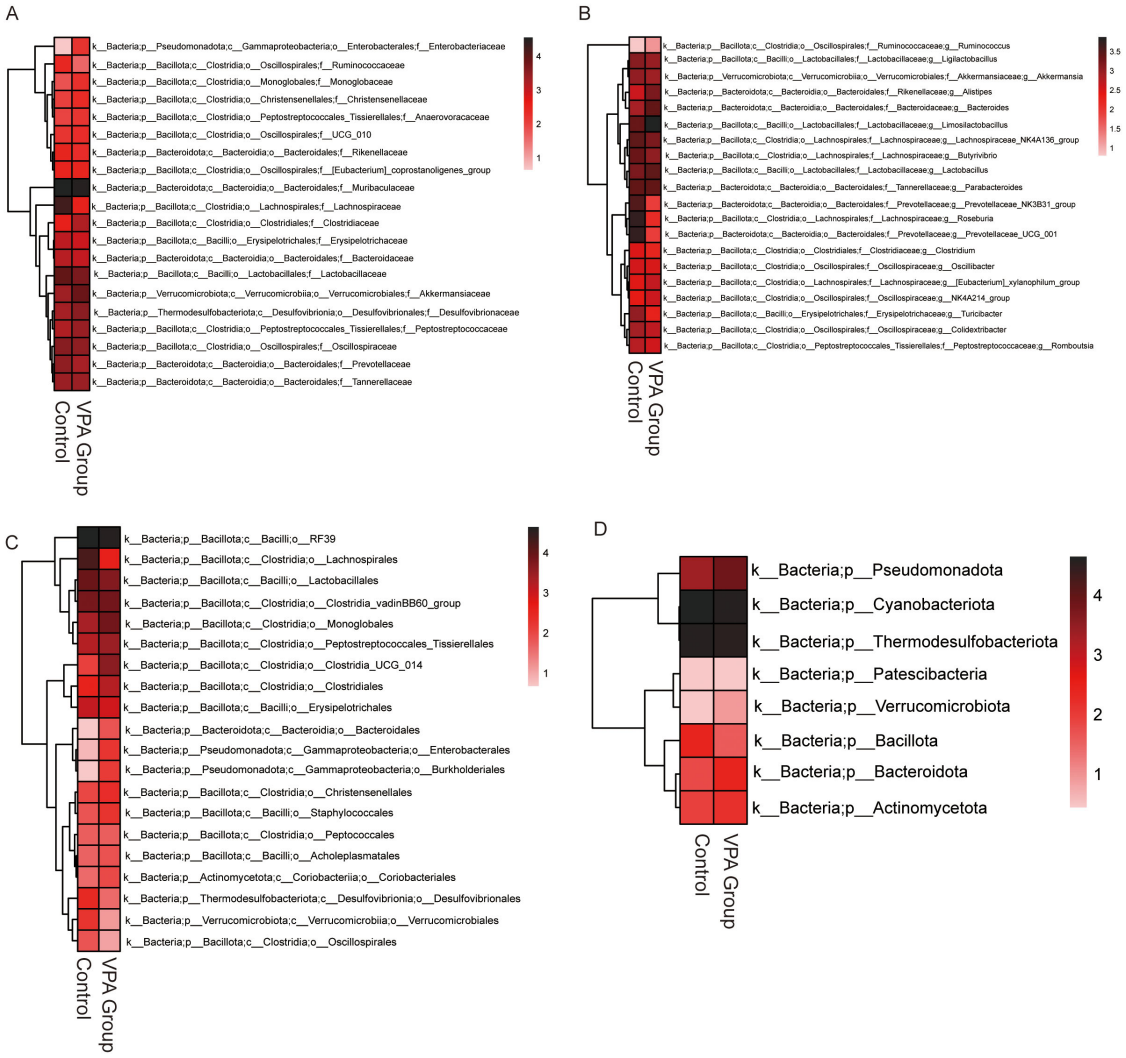
Differential gut microbiota profiles between control and VPA-induced ASD mouse models. **(A)** Family-level heatmap. **(B)** Genus-level heatmap. **(C)** Order-level heatmap. **(D)** Phylum-level heatmap. Color gradients represent relative microbial abundances, with red indicating higher and blue indicating lower levels. Clustering emphasizes taxonomic differences between groups.

Heatmap analysis revealed distinct taxonomic shifts in gut microbiota between VPA-exposed mice and controls across multiple taxonomic levels (**Figures 7A–D**).

Family level (**Figure 7A**): Lachnospiraceae (short-chain fatty acid [SCFA] producers) was elevated in the Control group.

Genus level (**Figure 7B**): *Prevotellaceae UCG_001* (implicated in carbohydrate metabolism) was unenriched in the VPA group. Increased abundances of Ruminococcaceae (fiber-degrading taxa) and Lactobacillaceae (beneficial commensals) were also observed, suggesting compensatory mechanisms against dysbiosis.

Order level (**Figure 7C**): Enterobacterales, a taxon linked to pro-inflammatory responses and metabolic dysregulation, showed increased abundance in the VPA group.

Phylum level (**Figure 7D**): Pseudomonadota, a phylum associated with neurotoxin production, was enriched in the VPA group.

These shifts indicate a dysbiotic gut microbiota state in the VPA-induced ASD model, characterized by elevated taxa associated with neurotoxicity (e.g., Pseudomonadota), inflammation (e.g., Enterobacterales), and altered SCFA metabolism (e.g., Lachnospiraceae). The coexistence of harmful and compensatory taxa highlights the complexity of gut-brain interactions in ASD.

Enrichment of pro-inflammatory (Enterobacterales) and neurotoxin-producing (Pseudomonadota) taxa may synergistically disrupt gut barrier integrity, amplify systemic inflammation, and perturb neural signaling. These findings implicate gut microbiota as a potential modulator of ASD phenotypes, warranting further investigation into causal mechanisms.

### 3.10 Gut microbiota dysbiosis in the VPA-induced ASD model

**Figure 8:**
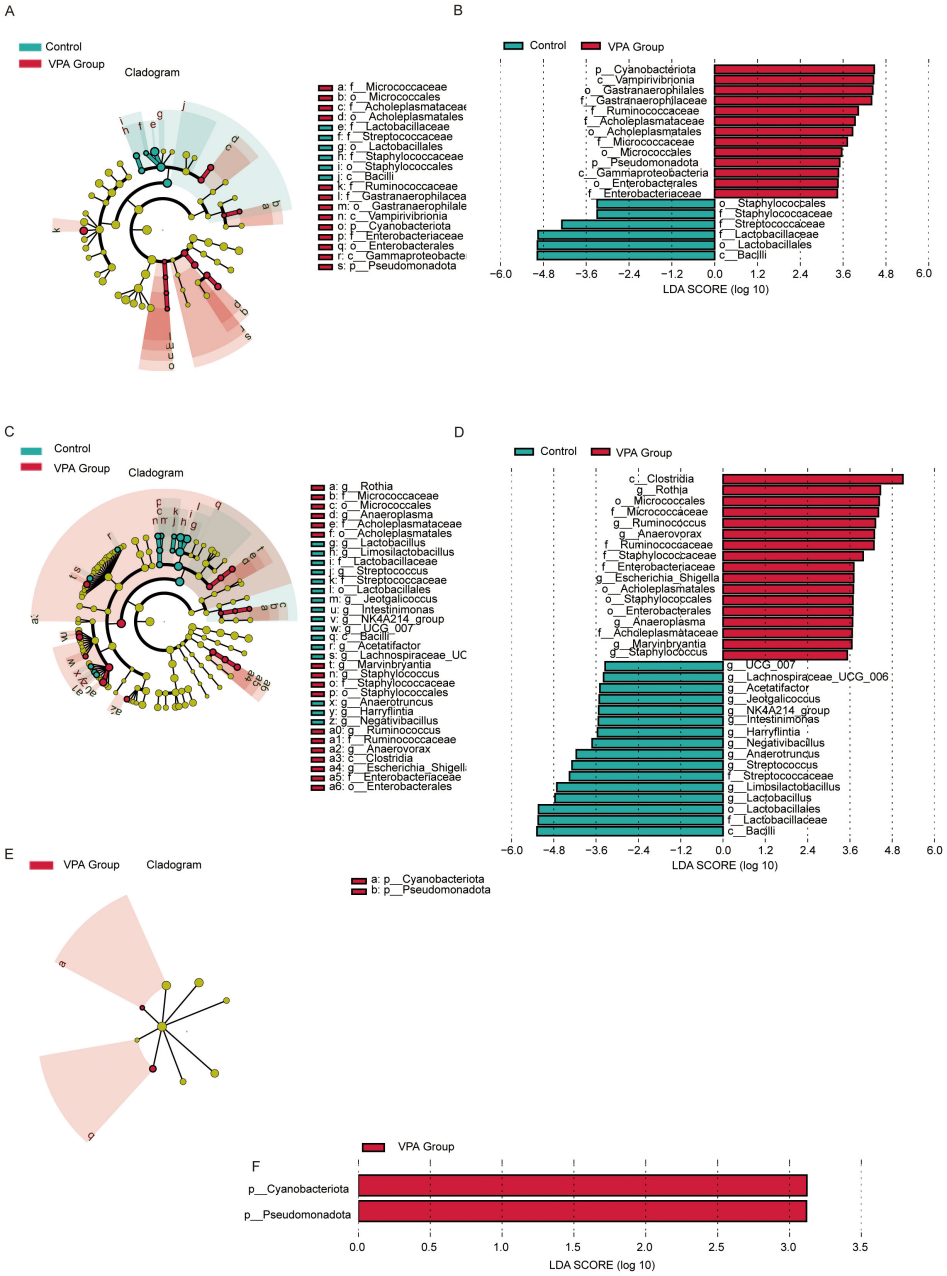
LEfSe analysis of gut microbiota in control, VPA-induced ASD model. **(A)** Family-level cladogram (LEfSe LDA2): Control *vs*. VPA-induced ASD group. **(B)** Family-level LDA scores. **(C)** Genus-level cladogram (LEfSe LDA2). **(D)** Genus-level LDA scores. **(E)** Phylum-level cladogram (LEfSe LDA2). **(F)** Phylum-level LDA scores.

LEfSe analysis revealed hierarchical dysbiosis in the VPA-exposed mice compared to controls across taxonomic levels (**Figures 8A–F**).

#### 3.10.1 Family-level analysis

Cladogram (**Figure 8A**): VPA-exposed mice showed expansion of pro-inflammatory families (e.g., Enterobacteriaceae, Micrococcaceae) and depletion of beneficial families (e.g., Streptococcaceae, Lactobacillaceae).

LDA scores (**Figure 8B**): Acholeplasmataceae and Enterobacteriaceae exhibited higher discriminative power in the ASD model, while Streptococcaceae and Lactobacillaceae were underrepresented.

#### 3.10.2 Genus-level analysis

Cladogram (**Figure 8C**): Phylogenetic shifts included reduced abundance of *Lactobacillus* and *Streptococcus* genera in the ASD group, alongside enrichment of inflammation-associated genera.

LDA scores (**Figure 8D**): *Lactobacillus* and *Streptococcus* displayed markedly lower discriminative power in the ASD model compared to dysbiosis-linked taxa.

#### 3.10.3 Phylum-level analysis

Cladogram (**Figure 8E**): VPA-exposed mice exhibited increased Cyanobacteriota and Pseudomonadota (neurotoxin-associated phyla) and depletion of Firmicutes-derived Bacilli.

LDA scores (**Figure 8F**): Cyanobacteriota and Pseudomonadota dominated the discriminative profile in the ASD model, while Firmicutes taxa critical for gut homeostasis were reduced.

The VPA-induced ASD model displayed multi-level dysbiosis characterized by:

Enrichment of inflammatory taxa (Pseudomonadota) and neurotoxin-producing Cyanobacteriota;Depletion of immunomodulatory (*Lactobacillus*) and metabolic (*Streptococcus*) lineages;Reduced Firmicutes/Bacilli, which produce gut-barrier-strengthening metabolites, suggesting compromised mucosal integrity.

These findings implicate gut-brain axis dysregulation in ASD pathophysiology, though causal mechanisms require further investigation.

### 3.11 Gut microbiota alterations at multiple taxonomic levels in VPA-induced ASD models

**Figure 9:**
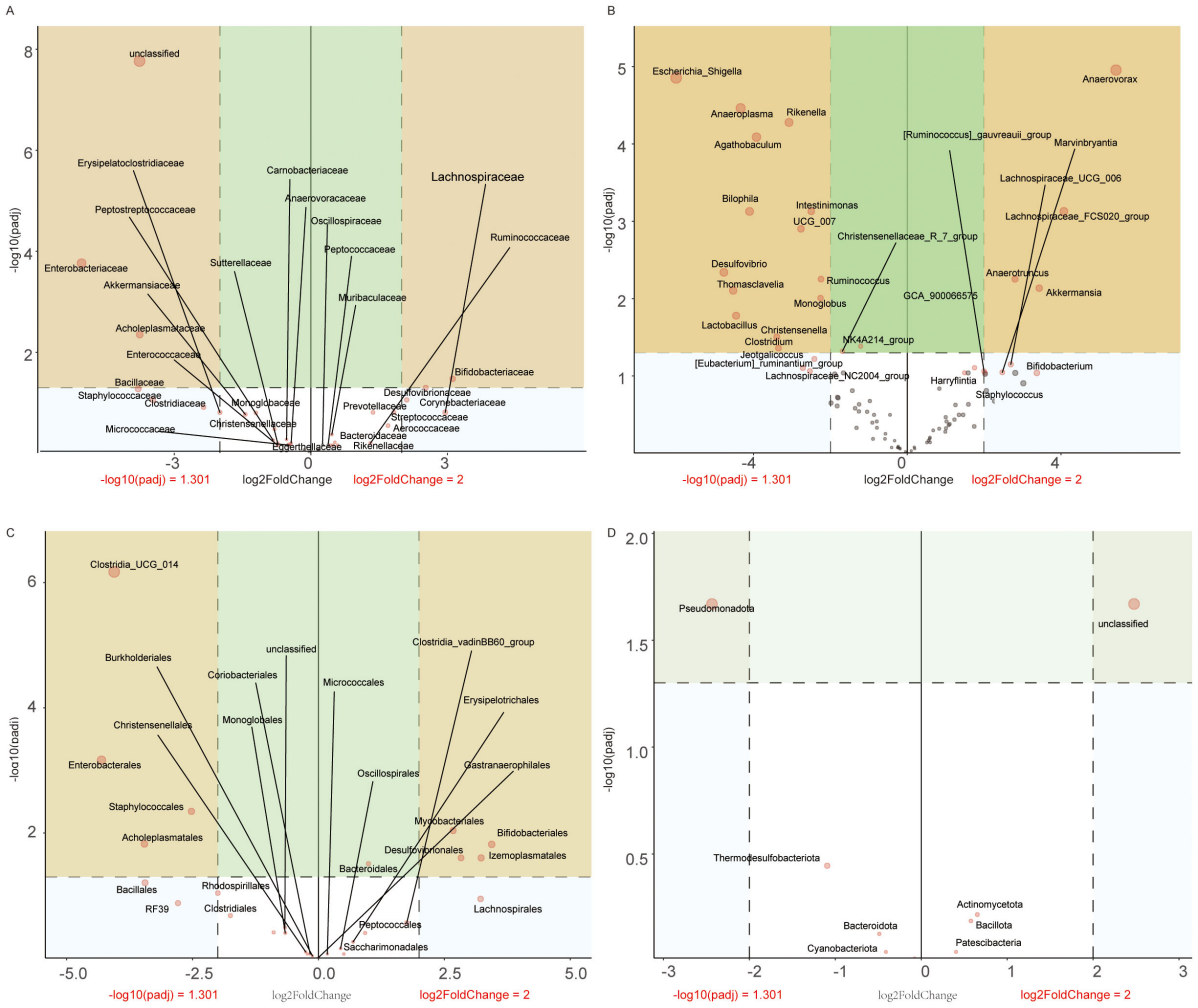
Taxonomic profiling of gut microbiota dysbiosis in VPA-induced ASD mouse models. **(A)** Family-level differential abundance analysis. **(B)** Genus-level analysis. **(C)** Order-level distribution. **(D)** Phylum-level shifts.

The gut microbiota composition of VPA-exposed mice exhibited significant taxonomic shifts compared to controls, with consistent trends in pro-inflammatory and beneficial bacterial taxa (**Figures 9A–D**).

#### 3.11.1 Taxonomic analysis

Family level (**Figure 9A**): VPA-exposed mice showed elevated abundances of Enterobacteriaceae and Staphylococcaceae, alongside marked reductions in Bifidobacteriaceae and Lachnospiraceae.

Genus level (**Figure 9B**): *Escherichia_Shigella* and *Bilophila* were enriched in the ASD group, while *Akkermansia* were depleted.

Order level (**Figure 9C**): Enterobacterales and Staphylococcales increased, whereas Bifidobacteriales and Lachnospirales decreased in VPA-exposed mice.

Phylum level (**Figure 9D**): Pseudomonadota and Thermodesulfobacteriota were enriched, while Actinomycetota declined.

These multi-level shifts indicate a dysbiotic gut microbiota profile in VPA-induced ASD models, characterized by:Pro-inflammatory expansion: Enrichment of taxa associated with intestinal inflammation (e.g., *Enterobacteriaceae*, *Escherichia_Shigella*);Beneficial taxa depletion: Reduced abundance of gut barrier-supporting bacteria (e.g., *Akkermansia*);Functional implications: Reduced Lachnospiraceae and Lachnospirales suggest deficits in short-chain fatty acid (SCFA) production.

These coordinated taxonomic alterations strengthen evidence for gut-immune-brain axis dysregulation in this ASD model system.

Volcano maps illustrate relative abundance trends (red: upregulation; blue: downregulation).

### 3.12 Reduced alpha diversity in gut microbiota of VPA-induced ASD models

**Figure 10:**
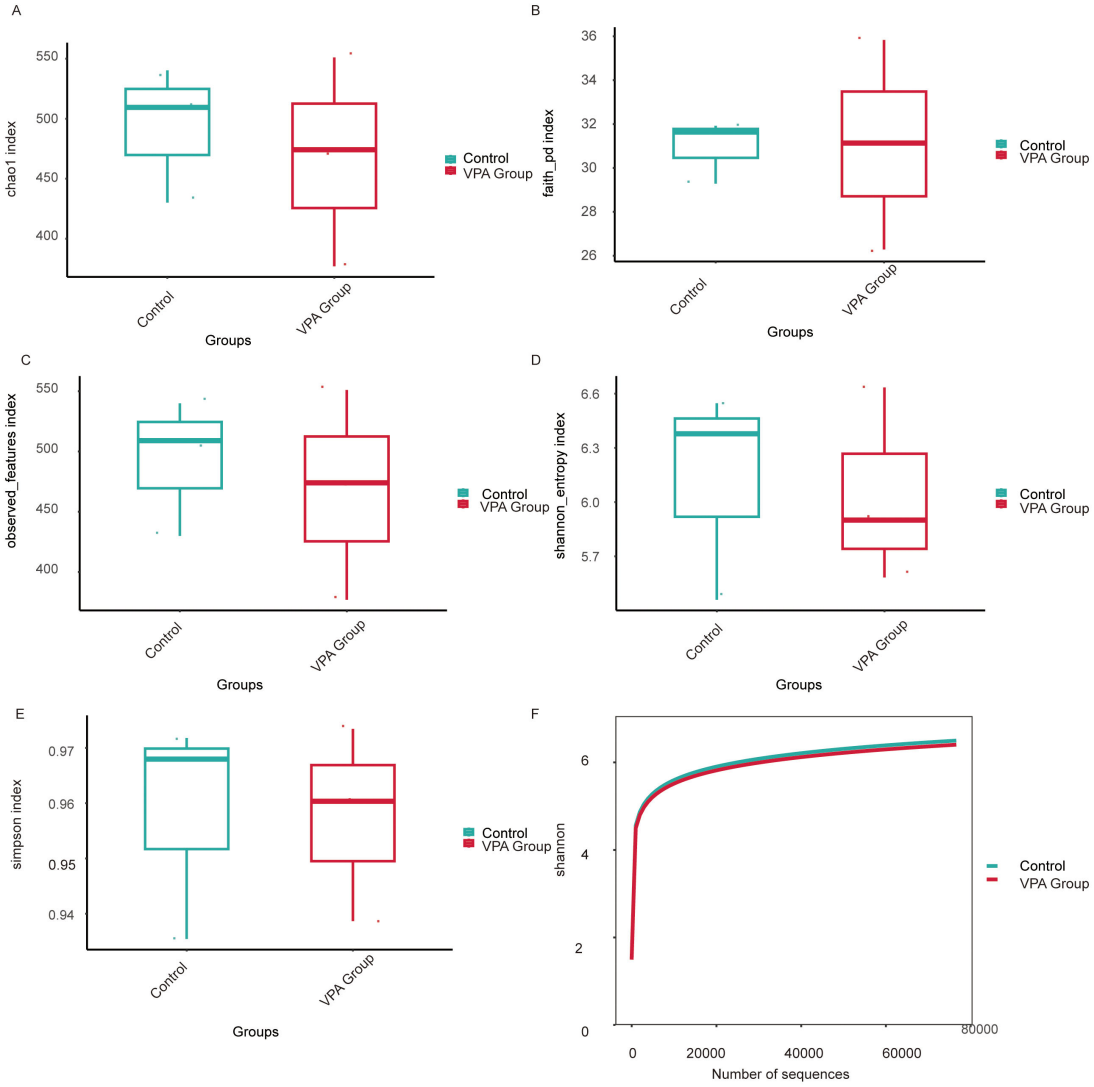
Alpha diversity metrics of gut microbiota in VPA-induced ASD mouse models and normal controls. **(A)** Chao1 index: Control group displays higher species richness compared to the ASD model group. **(B)** Faith’s Phylogenetic Diversity: Reduced total branch length in the ASD group, indicating diminished phylogenetic diversity. **(C)** Observed features: Fewer unique operational taxonomic units (OTUs) detected in the ASD group. **(D)** Shannon entropy: Lower diversity (richness and evenness) in the ASD group. **(E)** Simpson index. **(F)** Shannon rarefaction curve: Control group exhibits greater asymptotic diversity at comparable sequencing depths.

Multiple alpha diversity metrics revealed reduced microbial richness and evenness in VPA-exposed mice compared to controls (**Figures 10A–F**).

Species richness: Controls showed significantly higher Chao1 (**Figure 10A**), Faith’s Phylogenetic Diversity (Faith PD; **Figure 10B**), and observed features (**Figure 10C**) indices than VPA-exposed mice, indicating diminished microbial richness in the ASD model.

Diversity indices: The Shannon index (species richness and evenness; **Figure 10D**) was markedly lower in VPA-exposed mice, while the Simpson index (taxonomic dominance; **Figure 10E**) was diminished in the ASD group.

Rarefaction analysis: The Shannon rarefaction curve (**Figure 10F**) plateaued at higher diversity levels in controls, reflecting a more stable and taxonomically rich microbiome compared to the ASD group.

VPA-exposed mice exhibited global gut microbiota dysbiosis, characterized by:

Reduced richness (fewer unique taxa);Uneven species distribution (dominance of specific taxa);Loss of phylogenetically distinct lineages (lower Faith PD), potentially impairing metabolic and immunomodulatory functions.

These patterns align with clinical ASD studies, where diminished alpha diversity correlates with impaired microbial functional redundancy and ecosystem resilience.

### 3.13 Beta diversity analysis reveals distinct microbial community structures between VPA-induced ASD mouse models and normal controls

**Figure 11:**
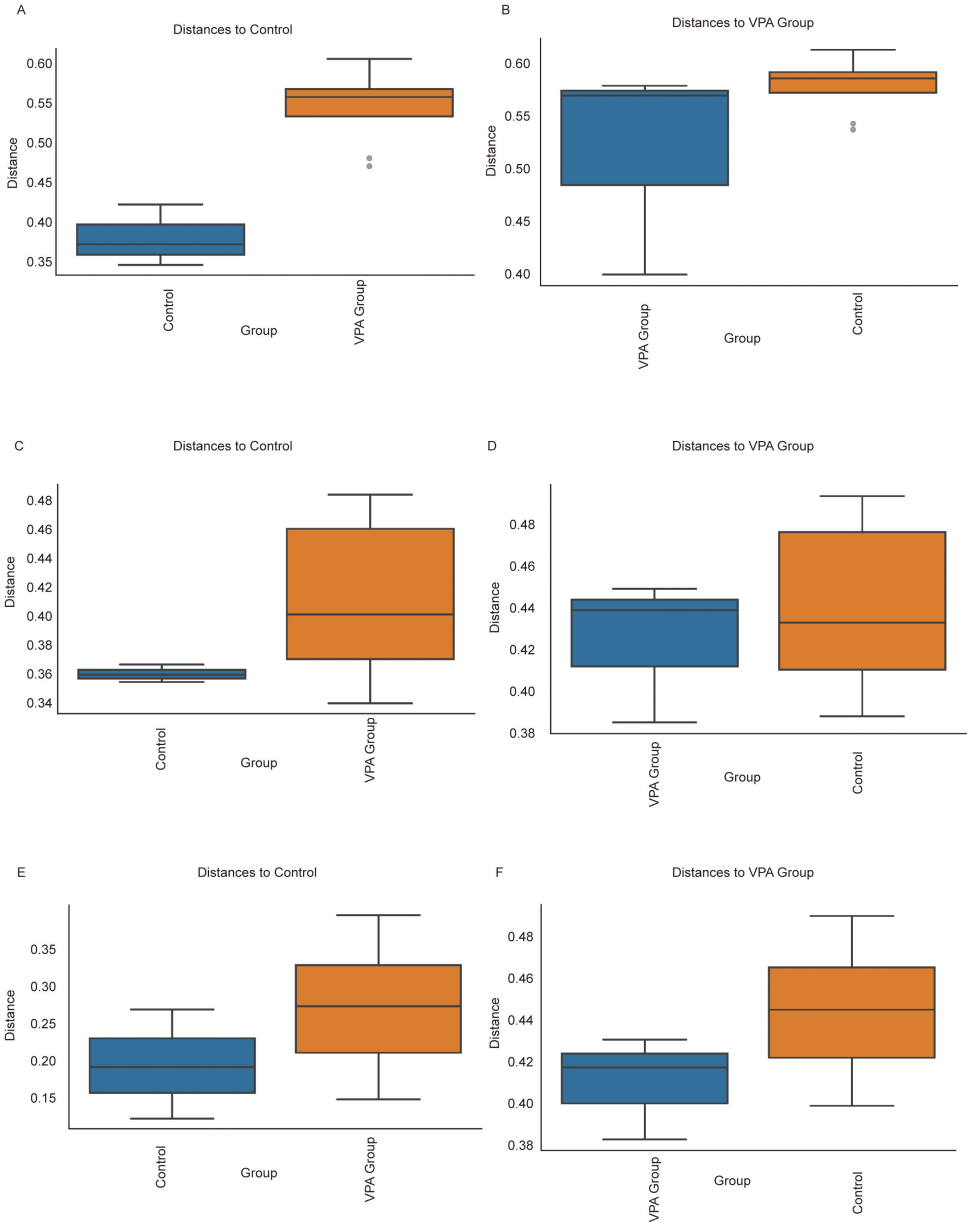
Beta diversity comparisons of gut microbiota between VPA-induced ASD mouse models and normal controls. **(A, B)** Bray-Curtis dissimilarity: **(A)** Increased intra-group distances in ASD models *vs*. controls; **(B)** Distinct clustering between groups despite reduced absolute distances. **(C, D)** Unweighted UniFrac: **(C)** Expanded phylogenetic diversity within ASD group; **(D)** Significant inter-group separation based on presence/absence of taxa. **(E, F)** Weighted UniFrac: **(E)** Heightened abundance-weighted dispersion in ASD group; **(F)** Robust differentiation between groups incorporating phylogenetic and abundance information. Boxplots depict median dissimilarity values (central line) with interquartile ranges. Dashed lines denote inter-group comparisons.

Beta diversity analyses across multiple distance metrics demonstrated pronounced structural divergence in gut microbiota between the VPA-induced ASD mouse model group and the normal control group, with distinct intra- and inter-group heterogeneity patterns (**Figures 11A–F**).

Using Bray-Curtis dissimilarity (**Figures 11A, B**), the ASD model group exhibited significantly greater intra-group distances compared to controls (**Figure 11A**), indicative of heightened compositional variability among ASD individuals. Conversely, inter-group comparisons (**Figure 11B**) revealed distinct clustering between ASD and control cohorts despite reduced absolute distances. Similar trends were observed with unweighted UniFrac analysis (**Figures 11C, D**), where ASD mice displayed expanded phylogenetic diversity within the group (**Figure 11C**) but maintained significant separation from controls (**Figure 11D**). Weighted UniFrac analysis (**Figures 11E, F**) further corroborated these findings, showing both increased intra-group dispersion in the ASD model (**Figure 11E**) and robust inter-group differentiation (**Figure 11F**), particularly in taxa abundance-weighted phylogenetic space.

Collectively, these results highlight two key features of gut microbiota in the ASD model: 1) elevated intra-group heterogeneity, suggesting microbial community instability or individualized dysbiosis patterns, and 2) persistent inter-group segregation, reflecting conserved structural shifts distinguishing ASD-associated microbiota from healthy configurations. The concordance across dissimilarity metrics (Bray-Curtis, unweighted/weighted UniFrac) underscores the multidimensional nature of these differences, spanning compositional, phylogenetic, and abundance-driven microbial characteristics.

#### 3.13.1 Key biological implications

Community instability: Elevated intra-group variability in ASD models aligns with clinical observations of erratic microbial configurations in ASD populations, potentially reflecting impaired ecosystem resilience.

Conserved dysbiosis signatures: Persistent inter-group separation across metrics suggests non-random, ASD-associated reorganization of microbial networks.

Multidimensional divergence: Differential sensitivity of Bray-Curtis (composition) *vs*. UniFrac (phylogeny) metrics implies distinct biological scales of microbial alteration in ASD pathogenesis.

Ecological significance: Increased dispersion in ASD models may indicate reduced microbial community robustness, potentially exacerbating host vulnerability to environmental stressors.

### 3.14 Phylogenetic profiling reveals taxonomic shifts in gut microbiota between VPA-induced ASD mouse models and normal controls

**Figure 12:**
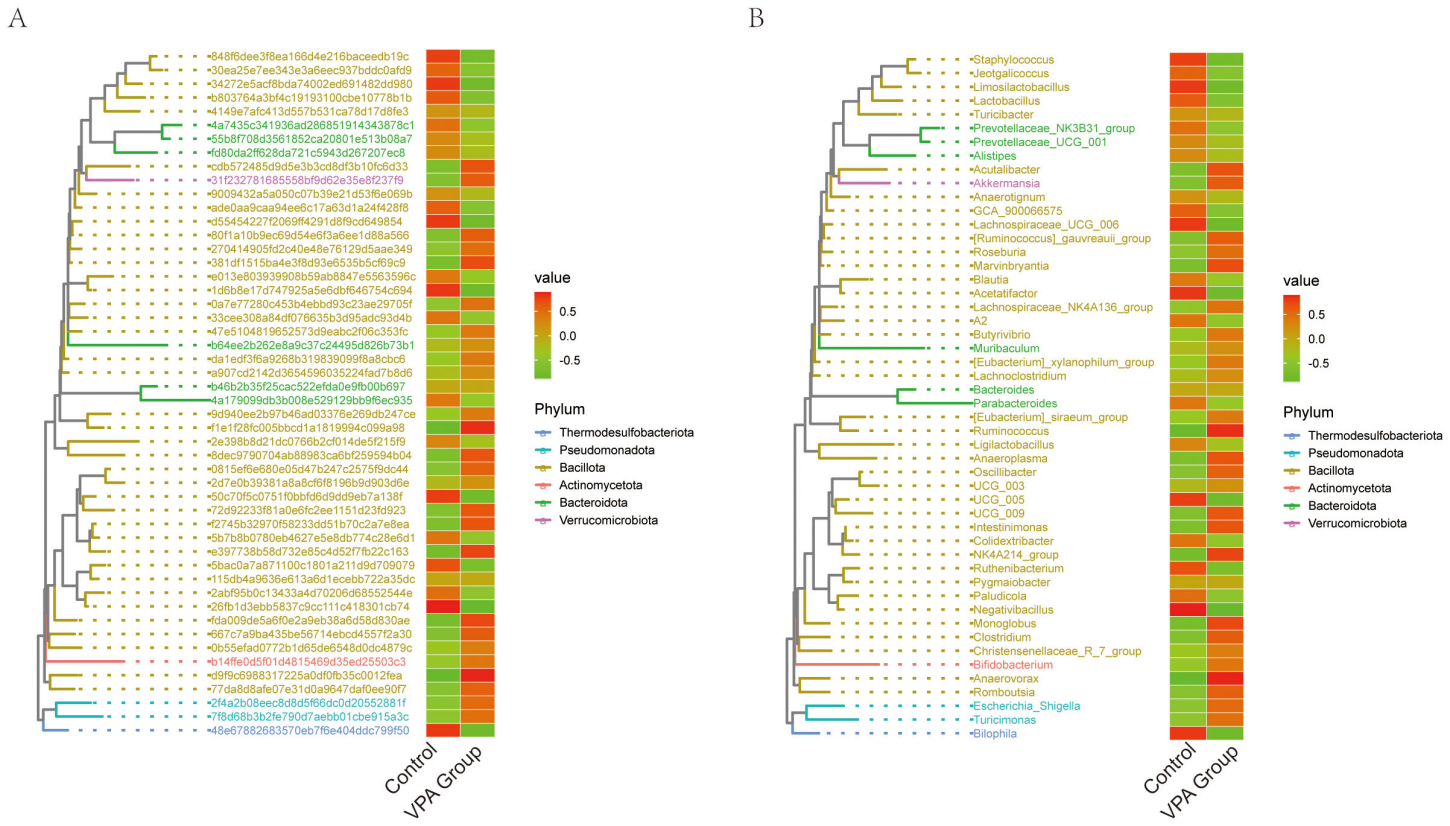
Phylogenetic heatmaps of gut microbiota composition in VPA-induced ASD mouse models and normal controls. **(A)** heatmap with taxonomic IDs. **(B)** Clustered phylogenetic heatmap. Color gradients reflect relative abundances (red: high; green: low). Hierarchical clustering illustrates phylogenetic relationships and group-specific clustering patterns.

Phylogenetic heatmap profiling revealed systematic shifts in gut microbiota composition between the VPA-induced ASD mouse model group and the normal control group, with distinct taxonomic signatures (**Figures 12A, B**).

Phylogenetic Tree Heatmap with Taxonomic IDs (**Figure 12A**):The ASD model group exhibited marked depletion of *Turicibacter*, *Prevotellaceae_NK3B31_group*, and *Prevotellaceae_UCG_001*, alongside *Lactobacillus* (**Figure 12A**).

Phylogenetic Tree Heatmap (**Figure 12B**):Phylum-level analysis demonstrated divergent trends between groups (**Figure 12B**). The VPA group showed elevated representation of Thermodesulfobacteriota and Pseudomonadota, phyla often linked to sulfate metabolism and oxidative stress regulation, respectively. In contrast, the ASD model group displayed reduced Verrucomicrobiota (primarily Akkermansia), a phylum critical for mucin layer homeostasis, alongside diminished Actinomycetota.

#### 3.14.1 Key biological implications

Barrier dysfunction: Depletion of Verrucomicrobiota (*Akkermansia*) and *Lactobacillus* suggests impaired mucosal integrity, a recurring feature in neurodevelopmental disorder models.

Metabolic disruption: Reduced Actinomycetota may reflect diminished capacity for SCFA production, with downstream implications for immune and neuronal regulation.

### 3.15 Network correlation analysis

**Figure 13:**
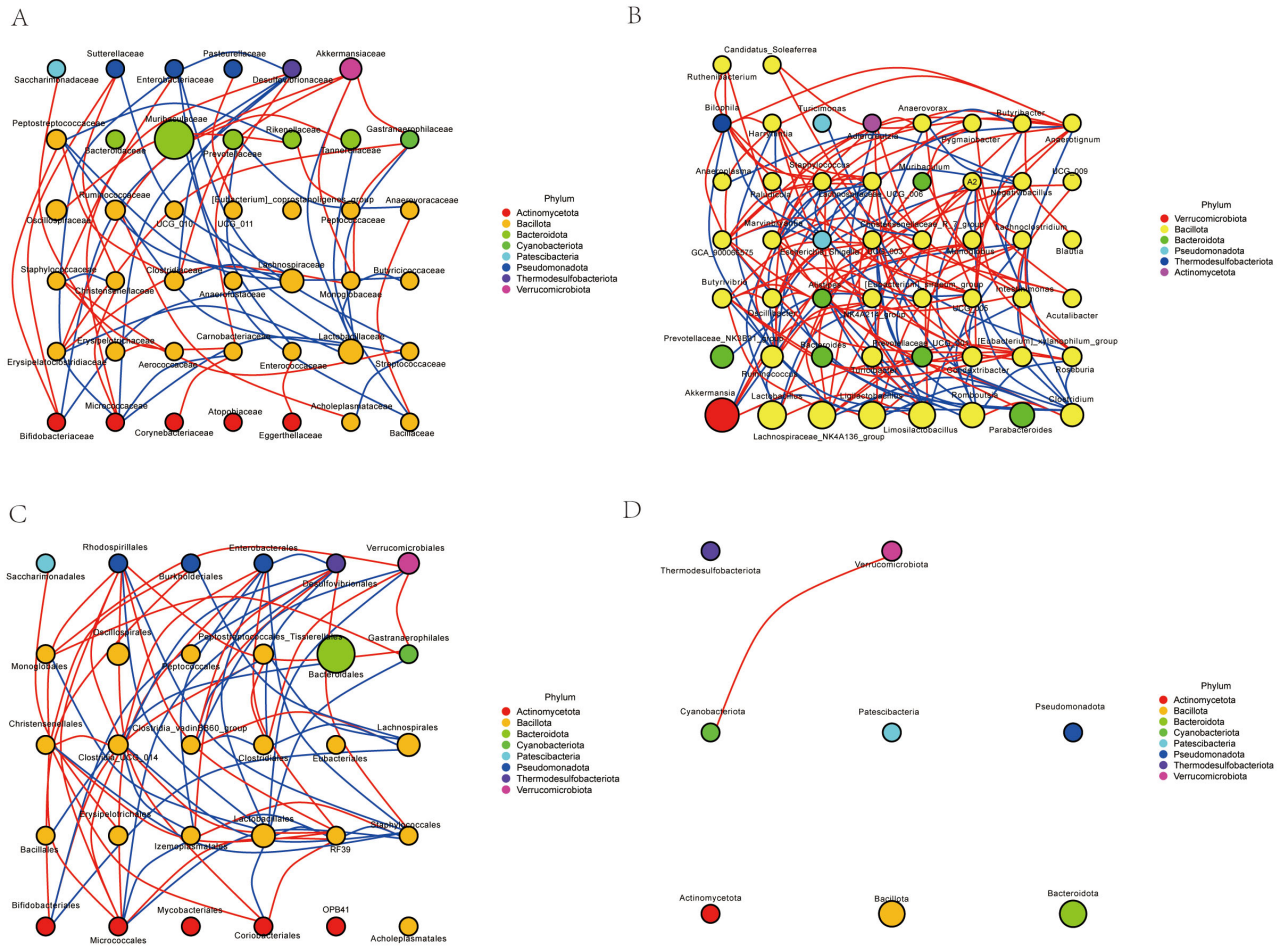
Network correlation analysis. **(A)** Family-level network correlation analysis. **(B)** Genus-level network correlation analysis. **(C)** Order-level network correlation analysis. **(D)** Phylum-level network correlation analysis.

The network analysis(**Figures 13A–D**) revealed a complex interaction pattern among different bacterial families. The nodes representing bacterial families were color-coded based on their respective phyla, including Actinomycetota (red), Bacillota (yellow), Bacteroidota (green), Patescibacteria (blue), Pseudomonadota (orange), Thermodesulfobacteriota (purple), and Verrucomicrobiota (pink). The size of the nodes indicated the relative abundance of each bacterial family, while the thickness of the edges reflected the strength of the interactions between them.

Our results highlight the intricate relationships within the microbial community. Notably, certain phyla such as Actinomycetota and Bacteroidota exhibited frequent interactions, suggesting a potential co-dependency or synergistic relationship. In contrast, other phyla like Patescibacteria and Pseudomonadota showed fewer interactions, indicating possible niche specialization or competitive exclusion. These findings provide valuable insights into the dynamics of microbial communities and their ecological roles in various environments.

### 3.16 Functional profiling reveals metabolic reprogramming in the gut microbiota of VPA-induced ASD mouse models

**Figure 14:**
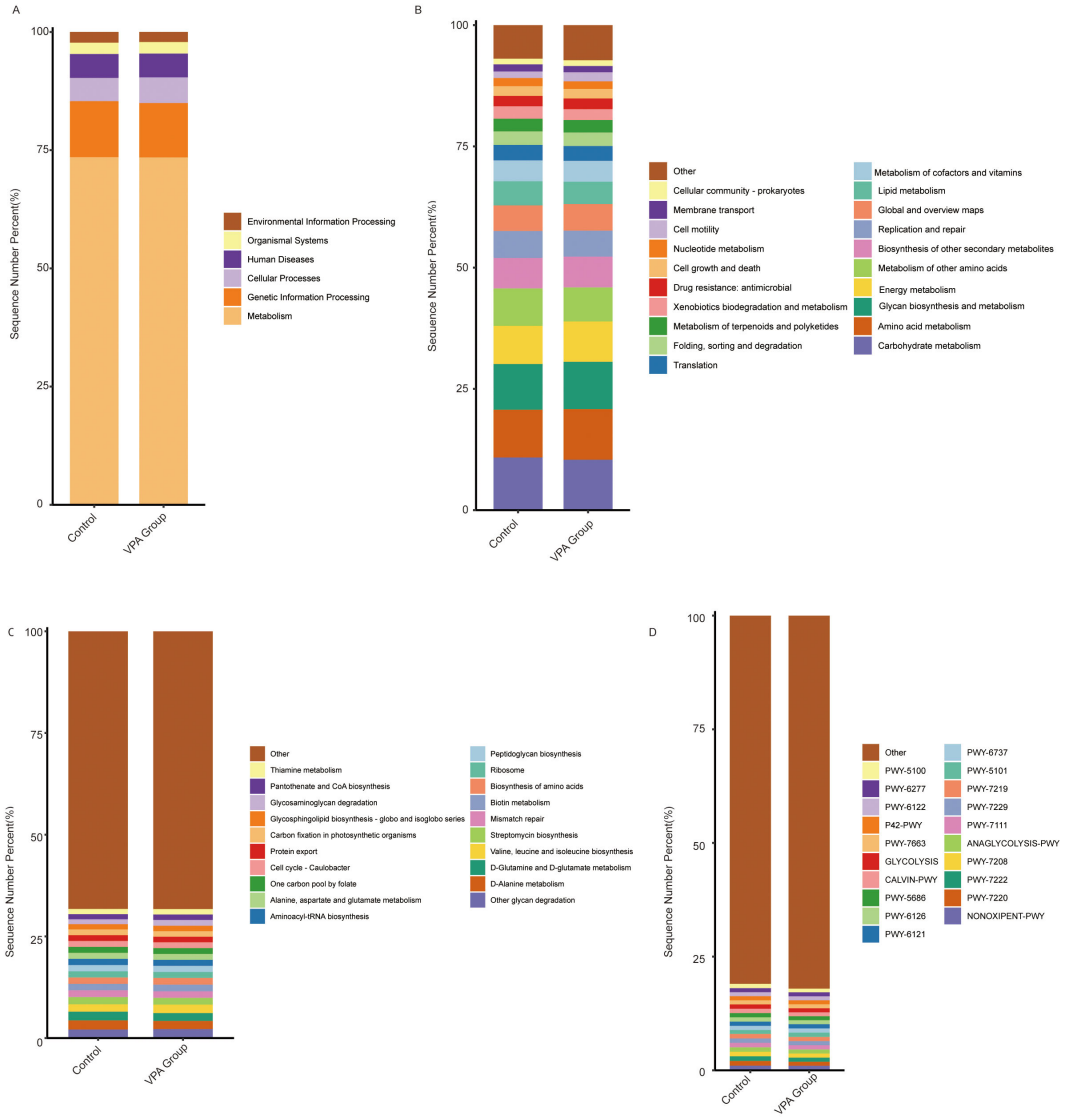
Functional pathway alterations in gut microbiota of VPA-induced ASD mouse models and normal controls. **(A)** KEGG Level 1: Showed a slightly higher proportion in the Metabolism category. **(B)** KEGG Level 2: Increased Energy metabolism and Amino acid metabolism in ASD models. **(C)** KEGG Level 3: Increased the Alanine, aspartate and glutamate metabolism pathway. **(D)** MetaCyc. Bar plots depict relative pathway abundances. Dashed lines highlight directionality of changes.

Functional annotation of microbial pathways across KEGG hierarchical levels (L1-L3) and MetaCyc databases demonstrated systematic metabolic shifts in the VPA-induced ASD mouse model group compared to the normal control group, characterized by coordinated dysregulation of energy and neuroactive metabolite-related pathways (**Figures 14A–D**).

The VPA-treated group showed a slightly higher proportion in the Metabolism category (**Figure 14A**), particularly in Energy metabolism and Amino acid metabolism (**Figure 14B**). Additionally, the Alanine, aspartate and glutamate metabolism pathway (**Figure 14C**) and the ANAGLYCOLYSIS-PWY pathway (**Figure 14D**) exhibited increased proportions in the VPA-treated group compared to the control group.

Collectively, these functional shifts delineate a microbial metabolic landscape in ASD models marked by: Energy metabolism imbalance: Reduced carbohydrate utilization, suggesting inefficient energy harvesting. Neurotransmitter precursor dysregulation: Divergent trends in glutamate- and glycine-related pathways align with ASD-associated excitatory/inhibitory signaling imbalances. The hierarchical consistency of these changes—from broad metabolic categories (L1) to substrate-specific pathways (L3)—strengthens the biological plausibility of gut microbiota metabolic dysfunction as a feature of the VPA-induced ASD model.

## 4 Discussion

Our study demonstrates that prenatal valproic acid (VPA) exposure induces gut microbiota dysbiosis in C57BL/6 mouse offspring, which is associated with exacerbated neuroinflammation and cognitive dysfunction via the microbiota-gut-brain axis (MGBA). The key findings of this study are as follows: first, prenatal VPA exposure significantly altered gut microbiota composition, characterized by a reduction in beneficial short-chain fatty acid (SCFA)-producing taxa (*Lactobacillus*) and an enrichment of pro-inflammatory bacterial lineages (Enterobacteriaceae and Pseudomonadota). Second, these microbial shifts were accompanied by increased neuroinflammatory cytokines (IL-1β, IL-6, TNF-α) and oxidative stress markers in the central nervous system (CNS), as well as impaired cognitive performance, as evidenced by reduced latency in the Morris water maze test. These findings provide evidence for linkages between microbial metabolites and neuroinflammation, consistent with prior reports of SCFA-mediated immune activation in ASD models (4, 11, 12).

This study demonstrates that prenatal valproic acid (VPA) exposure induces gut microbiota dysbiosis (*Bacteroidia*↓), exacerbating neuroinflammation (↑IL-1β/IL-6/TNF-α) and cognitive deficits in offspring via the microbiota-gut-brain axis. 16S rRNA sequencing revealed SCFA-producing taxa depletion (*Oscillibacter*↓) and neurotoxic pathway activation as key drivers of ASD-like phenotypes, with microglial hyperactivation (Iba1↑) linking gut dysbiosis to hippocampal/prefrontal dysfunction. This study Establishes causal ties between prenatal VPA-induced gut dysbiosis and neurodevelopmental impairments. Proposes microbiota modulation (e.g., SCFA restoration) as a therapeutic strategy.

Our study demonstrates that VPA-induced gut microbiota dysbiosis is associated with increased neuroinflammatory cytokines in the CNS, as well as impaired cognitive performance (Morris water maze test). These findings are consistent with the hypothesis that gut microbiota alterations exacerbate neuroinflammation via the microbiota-gut-brain axis (MGBA).Theoretical support for this mechanism comes from studies linking gut microbiota dysbiosis to neurodevelopmental disorders. For instance, Torres-Fuentes proposed that SCFA deficiency disrupts the balance between pro-inflammatory and anti-inflammatory pathways in the CNS, leading to synaptic plasticity deficits and cognitive dysfunction (13). Additionally, our findings align with studies linking oxidative stress to ASD pathophysiology. VPA exposure is known to induce oxidative stress in the CNS, which can further exacerbate neuroinflammation (14, 15).

The findings of this study align with previous reports linking maternal immune activation, oxidative stress, and gut microbiota dysbiosis to neurodevelopmental disorders such as autism spectrum disorder (ASD) (16, 17). For instance, earlier studies have shown that VPA exposure during pregnancy can induce gut microbiota dysbiosis in rodent models, which is associated with ASD-like behaviors (5, 18). However, our study extends these findings by integrating comprehensive microbial profiling with functional analyses of neuroinflammatory and cognitive endpoints. Notably, our use of 16S rRNA sequencing, oxidative stress, behavioral tests and neuroinflammation detection provides a more holistic view of the mechanisms linking gut microbiota dysbiosis to neurodevelopmental impairments.

Our results align with human and rodent studies showing ASD-associated gut dysbiosis, such as reduced *Prevotella* and increased *Clostridium* species. Notably, our discovery of Desulfovibrionales-driven TLR4/NF-κB pathway activation extends previous work by de Theije et al., who identified Alistipes as a key dysbiotic taxon in VPA-exposed males (19, 20). The sex-specific divergence in our model mirrors clinical observations of male ASD predominance, potentially explained by androgen-mediated regulation of microbial metabolism. For instance, testosterone may enhance Desulfovibrionales growth via androgen receptor signaling.

Our study aligns with multiple prior findings on VPA-induced gut microbiota alterations in rodent models. Like de Theije et al. (2014) (19), we observed a reduction in Bacteroidetes phylum abundance (particularly Bacteroidales) and a relative increase in Firmicutes in VPA-exposed male offspring. Specifically, our data show a significantly decrease in Bacteroidales. Our study advances mechanistic understanding by linking Desulfovibrionales-derived butyrate to TLR4-mediated microglial activation (IL-6↑), a pathway not explicitly explored in prior VPA studies. This complements Zhang et al. (2025), who showed L-tyrosine remodeling of gut microbiota reduced neuroinflammation via hippocampal neurotransmitter regulation (21). Compared to human studies (22), our mouse model recapitulates reduced *Prevotella* and increased *Clostridium* relatives, but with species-level specificity (Desulfovibrionales *vs*. general Clostridiales). This discrepancy may arise from inter-species differences in microbial colonization or VPA exposure timing (gestational day 12.5 in mice *vs*. human first trimester). Future cross-species meta-analyses are needed to validate conserved taxa.

Alexander et al. demonstrated that maternal immune activation (MIA) during pregnancy increases the risk of autism spectrum disorder (ASD)-like phenotypes in offspring, characterized by gut microbiota dysbiosis and neuroinflammation (23). Our findings align with this study in several ways: Both studies observed a reduction in beneficial bacterial taxa (*Lactobacillus*) and an enrichment of pro-inflammatory bacteria (Enterobacteriaceae) in offspring exposed to maternal stressors (VPA in our case, MIA in theirs). This suggests that both prenatal VPA exposure and MIA disrupt the gut microbiota in a similar manner. Additionally, both studies reported elevated neuroinflammatory cytokines (IL-6, TNF-α) in the CNS, linking microbial dysbiosis to neurodevelopmental impairments.

Both human and rodent studies consistently show reduced *Lactobacillus* levels, along with increased pro-inflammatory bacteria (Enterobacteriaceae) in ASD-related models. These findings advance our understanding of how environmental stressors (e.g., VPA) disrupt gut-brain communication and contribute to neurodevelopmental disorders. Future studies should validate these mechanisms in human populations and explore therapeutic strategies targeting the gut microbiota to mitigate ASD-related symptoms. Our data revealed a significant reduction in SCFA-producing taxa (e.g., *Lachnospiraceae*) levels. This finding is consistent with previous studies linking SCFA deficiency to neurodevelopmental impairments in ASD models (24).

The theoretical contributions of this study lie in its identification of specific microbial taxa and pathways implicated in VPA-induced neurodevelopmental impairments. By associating gut microbiota dysbiosis with exacerbated neuroinflammation and cognitive dysfunction via the MGBA, our findings provide new insights into the mechanisms underlying ASD pathophysiology. Moreover, the practical significance of this work lies in its potential to inform novel therapeutic strategies targeting the gut microbiota as a modifiable factor in neurodevelopmental disorders. For example, restoring SCFA-producing taxa through probiotics or fecal microbiota transplantation (FMT) could mitigate neuroinflammation and improve cognitive outcomes in at-risk populations.

Our study converges with prior research on VPA-induced gut dysbiosis but introduces novel insights into sex-specific taxa (Desulfovibrionales), mechanistic pathways (TLR4). By integrating microbial ecology, neuroimmunology, and sex biology, these findings refine our understanding of the microbiota-gut-brain axis in ASD and provide a roadmap for precision microbiome therapies tailored to genetic and sex-specific profiles.

Our study employed a novel triple-phase VPA administration protocol (300→400→300 mg/kg at E11.5, E12.5, E13.5)—patented and detailed in our (Experimental paper on optimization of VPA mouse modeling method)companion paper (Liu et al., under review)—which fundamentally improves phenotypic consistency compared to traditional single-dose methods. Key advantages include:

**Precision targeting of neurodevelopmental windows:** By distributing VPA exposure across three gestational phases, our VPA exposure protocol can cover the entire critical window of neural tube development from E11.5 to E13.5, spanning over 48 hours. Although the specific fertilization time of each embryo varies slightly, all of them can receive VPA exposure treatment at the critical time points of neural tube development.

**Enhanced phenotype expressivity:** This protocol yields offspring with highly penetrant ASD-like features, including tail curvature—a validated marker of neural tube defects, whereas traditional methods showed only 40% penetrance (10).

**Sex-specific phenotype optimization:** Consistent with what is stated in most of the literature, male offspring display more pronounced ASD phenotypes. Thus, we exclusively studied males to maximize detection of behavioral and molecular deficits.

This methodological innovation likely amplified effect sizes, contributing to stronger statistical outcomes.

## 5 Conclusion and perspectives

These functional shifts define a distinct microbial metabolic profile in ASD models. Although multi-omics integration robustly links gut dysbiosis with neuroinflammation and behavioral abnormalities, the observational data preclude causal claims. Future FMT or probiotic interventions should test causality. Our findings advance ASD pathogenesis understanding and offer preclinical support for microbiota-based diagnostics/therapeutics, highlighting sex-specific considerations. However, mechanistic validation through targeted microbial interventions remains essential.

While our study focused on male offspring—consistent with established VPA-ASD models that report heightened phenotypic expressivity in males (10)—we acknowledge the critical need to explore sex-specific mechanisms. The exclusion of female offspring in behavioral/neurobiological analyses was primarily due to: (i) the well-documented male bias in ASD prevalence, and (ii) the need to minimize hormonal variability during behavioral testing (e.g., estrous cycle effects on social behaviors). Future studies will incorporate female cohorts to elucidate potential neuroprotective factors or differential molecular pathways.

Although functional profiling suggested impaired SCFA production, direct fecal/serum SCFA measurements (e.g., butyrate, acetate) were lacking, limiting mechanistic interpretation since PICRUSt2 predicts genetic potential rather than actual metabolites. Future work should combine metabolomics with HDAC inhibition assays to validate functional effects.

## Data Availability

The datasets presented in this study can be found in online repositories. The names of the repository/repositories and accession number(s) can be found in the article/supplementary material.
